# Maternal exposure to bioclimatic stress and hypertensive disorders of pregnancy in Western Australia: identifying potential critical windows of susceptibility

**DOI:** 10.1007/s11356-024-34689-6

**Published:** 2024-08-15

**Authors:** Amanuel T. Gebremedhin, Sylvester Dodzi Nyadanu, Ivan C. Hanigan, Gavin Pereira

**Affiliations:** 1 Curtin School of Population Health, Curtin University, Kent Street, PerthBentley, WA 6102 Australia; 2 Education, Culture, and Health Opportunities (ECHO) Ghana, ECHO Research Group International, Aflao, Ghana; 3 WHO Collaborating Centre for Climate Change and Health Impact Assessment, Faculty of Health Science, Curtin University, Bentley, WA Australia; 4 enAble Institute, Curtin University, Perth, Kent Street, Bentley, WA 6102 Australia

**Keywords:** Temperature, Universal Thermal Climate Index, Heat stress, Cold stress, Gestational hypertension, Preeclampsia

## Abstract

The anthropogenic climate change may impact pregnancy outcomes. Rather than ambient temperature, we aimed to use a composite bioclimatic metric (Universal Thermal Climate Index, UTCI) to identify critical susceptible windows for the associations between bioclimatic exposure and hypertensive disorders of pregnancy (HDPs) risk. Daily UTCI exposure from 12 weeks of preconception through pregnancy was linked to 415,091 singleton pregnancies between 1st January 2000 and 31st December 2015 in Western Australia. Adjusted weekly-specific and cumulative odds ratios (ORs) and 95% confidence intervals (CIs) of gestational hypertension and preeclampsia were estimated with distributed lag non-linear and standard non-linear logistic regressions. Exposures from early pregnancy to week 30 were associated with greater odds of HDPs with critical susceptible windows, particularly elevated at the 1st (10.2 °C) and 99th (26.0 °C) exposure centiles as compared to the median (14.2 °C). The most elevated ORs were 1.07 (95% CI 1.06, 1.08) in weeks 8–18 for gestational hypertension and 1.10 (95% CI 1.08, 1.11) in weeks 11–16 for preeclampsia for the 99th exposure centile. Cumulative exposures associated with HDPs with relatively higher but less precise ORs. The effects of high exposure to HDPs indicated sociodemographic inequalities. The identified critical periods and subpopulations could benefit from climate-related interventions.

**Supplementary Information:**

The online version contains supplementary material available at 10.1007/s11356-024-34689-6.

## Introduction

The increasing anthropogenic climate change crisis has set the global stage for new devastating public health threats (Intergovernmental Panel on Climate Change 2021). The effect on reproductive and fetal health is particularly worrying as this could have both present and generational adverse implications (Giudice et al. 2021; Segal and Giudice 2022). Previous studies on extreme temperature and pregnancy outcomes mainly focused on birth outcomes such as preterm birth, stillbirth, and low birth weight and mostly found positive associations (Nyadanu et al. 2024; Dalugoda et al. 2022). Related epidemiologic evidence for pregnancy complications such as hypertensive disorders of pregnancy (HDPs) is limited. HDPs which consist of gestational hypertension and preeclampsia are frequently reported pregnancy complications, occurring in 4–10% of pregnancies (Antza et al. 2022; Umesawa and Kobashi 2017). HDPs are major contributors to many maternal morbidities (e.g., cardiovascular and renal diseases), adverse birth outcomes, and maternal and fetal mortality (Antza et al. 2022; Wu et al. 2020). Risk factors of HDPs include several modifiable factors (e.g., smoking and anemia) and non-modifiable factors (e.g., maternal age, genetic or hereditary, previous pregnancy complications) (Umesawa and Kobashi 2017). However, the prediction of HDPs using the various risk factors and the search for preventive or therapeutic measures are still yet to be achieved as biological mechanisms are poorly understood (Umesawa and Kobashi 2017).

Recent epidemiologic studies have shown that maternal exposure to extreme ambient temperatures is a potential major modifiable risk factor for HDP (Mao et al. 2023). Ambient temperature could contribute to the risk of HDP through thermally-induced oxidative stress that affects placental development and physiology (Samuels et al. 2022; Nyadanu et al. 2024). Given increasing climate change concerns, further studies have been suggested to understand the impacts and critical susceptible windows of extreme temperatures on pregnancy complications and biological mechanisms to develop appropriate clinical and preventive strategies (Dalugoda et al. 2022; Giudice 2021; Giudice et al. 2021; Samuels et al. 2022; Nyadanu et al. 2024). Most of the previous studies either examined short-term effects (Khodadadi et al. 2022; Qu et al. 2021; Zhao et al. 2022) or cumulative exposures over longer periods such as trimesters (Auger et al. 2017; Shashar et al. 2020; Xiong et al. 2020). Assessment of long-term rather short-term effects is more appropriate because HDP diagnosis is usually made at discrete time points of convenience such as during antenatal care visits or at the onset of severe symptoms potentially delayed after disease onset (Mao et al. 2023; Xiong et al. 2020). Thus, the exact date of HDPs is not known and HDPs cannot be considered acute outcome events for short-term effects analysis (Mostofsky et al. 2018). This issue can be minimized by the assessment of longer-term exposures such as trimester averages, but such an approach removes the ability to identify fine time windows of susceptibility. Also, it has been demonstrated that effect estimates from trimester-average exposures could be biased with incorrect identification of critical windows (Wilson et al. 2017). A more appropriate method is long-term effect analysis with a distributed lag non-linear model (DLNM) that simultaneously captures both the intensity and timing of past exposures at finer temporal scales that may be biologically relevant but do not correspond to the clinically pre-defined trimesters (Gasparrini 2014; Gasparrini et al. 2010; Wilson et al. 2017). Although no method can “fix” the issue of uncertain time of HDP onset, the DLNM accounts for the temporal correlation of effects and may therefore better elucidate critical windows of exposure as compared to trimester-average exposures (Gasparrini 2014; Wilson et al. 2017).

Four studies have adopted DLNM to examine weekly ambient temperature exposure and HDPs (Part et al. 2022; Qian et al. 2023; Youssim et al. 2023; Zeng et al. 2024). High temperatures in early pregnancy were associated with a higher risk of severe HDPs, but tended to be “protective” in mid-late pregnancy (Part et al. 2022), at the beginning and late gestation weeks with a higher risk of preeclampsia (Youssim et al. 2023), and a higher and lower (protective) risk of preeclampsia in early and late pregnancy periods, respectively (Zeng et al. 2024). The fourth study found both low and high temperatures were associated with a lower risk of gestational hypertension (Qian et al. 2023). This requires further investigation, preferably with a composite biothermal or bioclimatic (hereafter bioclimatic) exposure metric as human thermophysiology is a function of multiple climatic factors and physiological processes that cannot be captured adequately with only air or apparent temperature (Matzarakis 2021; Staiger et al. 2019; Vanos et al. 2020). Several recent recommendations have been made to use composite bioclimatic metrics that include the total thermal environment (air and radiant temperatures, humidity, and wind) and physiological processes to obtain more robust and realistic thermal-related health estimates and risk projections (Matzarakis 2021; Nazarian and Lee 2021; Staiger et al. 2019; Vanos et al. 2020). A recent comprehensive evaluation of several thermal metrics recommended four principal bioclimatic metrics (Staiger et al. 2019). Among them, the Universal Thermal Climate Index (UTCI) derived from the advanced Fiala multi-node model of human thermoregulation with relatively high suitability and simulation of the climatic sensitivity of the human body (Blazejczyk et al. 2013; Bröde et al. 2012; Jendritzky et al. 2012) has been used in many thermal-health outcomes, warning, and forecasting studies as reviewed elsewhere (Krüger 2021; Romaszko et al. 2022). One previous study on the topic used UTCI but derived UTCI from climatic variables from only one synoptic meteorological station and investigated only short-term effects on HDPs with DLNM (Khodadadi et al. 2022). We have recently used spatiotemporal UTCI (Di Napoli et al. 2021) to investigate its association with birth outcomes (Nyadanu et al. 2023a, 2023b, 2022a, 2022b).

This study aimed to conduct an individual-level long-term analysis of the association between space–time varying weekly and cumulative exposure to UTCI and HDP to identify potential critical windows of susceptibility.

## Methods

### Study design, data sources, and population

An individual-level population-wide retrospective cohort study was conducted using births from 1st January 2000 to 31st December 2015 in the Western Australian Midwives Notification System (MNS) and Hospital Morbidity Data Collection (HMDC). MNS is a statutory routine data collection system that includes all births with ≥ 20 completed gestational weeks or ≥ 400 g fetal weight if the gestational length is not known (Government of Western Australia 2021). HMDC includes all hospitalization for pregnancy complications diagnoses according to the 9th/10th revision of the International Classification of Diseases and Australian Modification (ICD-9 and ICD-10-AM) diagnostic code. MNS and HMDC were linked by the Data Linkage Services of the Western Australia Department of Health via probabilistic matching of patient names and other identifiers as described elsewhere (Holman et al. 1999). The de-identified linked dataset contains sociodemographic and clinical information on both mother and baby, including maternal residential address as statistical area level 1 (SA1) at the time of birth delivery. SA1 is the second smallest geographical unit in Australia with a variable geographic size of a median of 19 hectares, each containing an average population of 400 people (Australian Bureau of Statistics 2011). The cohort included 415,091 singleton pregnancies of 20–42 completed gestational weeks by mothers ≤ 45 years old with SA1 addresses that were systematically selected from 474,835 pregnancies (Figure S1). To remove fixed cohort bias, pregnancies with conception dates < 20 weeks before the beginning of the cohort and > 42 weeks before the cohort ended were also excluded (Neophytou et al. 2021; Strand et al. 2011b).

### Outcomes

The outcomes of interest were HDPs that included gestational hypertension and preeclampsia. Gestational hypertension is defined as the new onset of hypertension (≥ 140/90 mmHg) after the 20th week of gestation (Antza et al. 2022). Preeclampsia is gestational hypertension that is accompanied by proteinuria or other end-organ damage such as to the liver or brain in a previously normotensive woman (Antza et al. 2022; Phipps et al. 2019). In this study, the HDPs were ascertained from the HMDS with the ICD-9 through to ICD-10-AM diagnostic codes as gestational hypertension (ICD-9:642.3, ICD-10: O13) and preeclampsia (ICD-9:642.4, 642.5, 642.7, ICD-10-AM: O14, O11). The definitions and diagnosis of hypertensive disorders of pregnancy were based on the Australasian Hypertension in Pregnancy Consensus Statement (Brown et al. 2000; Government of Western Australia 2021).

### Covariables

Covariables were selected a priori as covariates or potential confounders based on biological and epidemiological evidence in the literature (Auger et al. 2017; Nyadanu et al. 2023a; Part et al. 2022; Shashar et al. 2020; Xiong et al. 2020; Youssim et al. 2023). These included baby sex (male or female), the season of conception as a calendar month (1 to 12), index year of conception from 1999 to 2015 (1 to 17), marital status (married or unmarried), race or ethnicity (Caucasian or non-Caucasian), maternal age in years, parity (nulliparous or multiparous), smoking during pregnancy (smoker or non-smoker), and remoteness indicator (urban or non-urban). Maternal smoking status was self-reported in the birth record as yes (smoker) or no (non-smoker) if the woman smoked tobacco at any time during pregnancy. Tertiles of the area-level Index of Relative Socio-economic Disadvantage at local government areas derived by the Australian Bureau of Statistics census were used to define socioeconomic status (SES) as high, moderate, and low (Nyadanu et al. 2023a).

### Exposure

The primary exposure, UTCI is an equivalent air temperature (˚C) of the reference condition that captures both atmospheric heat exchanges with the human body (stress) and the body’s thermophysiological response (strain) (Blazejczyk et al. 2013; Bröde et al. 2012; Jendritzky et al. 2012). UTCI is a bioclimatic index derived from multiple meteorological factors (ambient temperature, humidity, wind speed, and solar radiation) and non-meteorological variables (biophysical properties of clothing and metabolic rate) under reference conditions. The reference conditions are 4 km/h walking speed, metabolic heat production of 2.3 MET (≃ 135 W m^−2^), a wind speed of 0.5 m/s at 10 m above the ground, mean radiant temperature equal to air temperature, and relative humidity of 50% or constant vapor pressure of 12 hPa (relative humidity was capped at a vapor pressure of 20 hPa for air temperature > 29 °C) (Bröde et al. 2012; Fiala et al. 2012). UTCI was developed based on the most advanced Fiala multi-node models of human thermoregulation (Blazejczyk et al. 2013; Bröde et al. 2012; Jendritzky et al. 2012). UTCI has ten standard categories with the range from + 9 °C to + 26 °C defined as *no thermal stress* while values below and above this range define cold and heat stresses, respectively, with increasing intensity up to extreme cold and heat stresses (Blazejczyk et al. 2013). It is worth noting that UTCI was not derived specifically for pregnant women, but to our knowledge modifications in heat or thermal indexes have not been made specifically for pregnancy. However, several recent medical, epidemiological, and thermal-health forecasting studies have applied UTCI as reviewed elsewhere (Krüger 2021; Romaszko et al. 2022). Di Napoli et al. recently provided a historical hourly temporal resolution in a 0.25° × 0.25° grid at a global scale of UTCI values as ERA5-HEAT dataset and publicly available at the Copernicus Climate Data Store (Di Napoli et al. 2021). We obtained 24-h averages of the gridded UTCI between 1st January 1999 to 31st December 2015 and processed at the SA1 levels across Western Australia using ArcGIS software (version 10.8.1). Daily UTCI exposure was assigned from 12 weeks before conception (Ha et al. 2017; Keikha et al. 2022; Nachman et al. 2016; Nyadanu et al. 2023a; Xiong et al. 2020) until either the day of diagnosis of HDPs for cases or birth for controls based on the dates and SA1 of the residential address. Seven-day averages for weekly exposure and cumulative exposures of preconception to HDPs event or birth, preconception, pregnancy, and three trimesters (weeks 1–13, 14–26, and 27-diagnosis or birth) were also estimated.

### Statistical analyses

To estimate the association between mean weekly UTCI and HDPs, we used DLNM logistic regression. A *cross-basis* matrix for DLNM was defined to account for the non-linear time-varying effects of the exposure from 12 weeks before conception through to 42 gestational weeks. In the cross-basis matrix, natural splines with 4 degrees of freedom (*df*) for exposure–response and 3 *df* for exposure period-response associations were specified. The optimal *df*s were chosen by testing several combinations of 2–7 *df* for the lowest Akaike Information Criterion (AIC) (Gasparrini 2014, 2021; Gasparrini et al. 2010) and visual inspection (Perperoglou et al. 2019). Using median exposure as a reference, we estimated odd ratios (ORs) and 95% confidence intervals (CIs) of HDPs for exposures at the 1st, 5th, 95th, and 99th centiles of weekly UTCI exposure. Separate models were also fitted to examine how UTCI exposure was associated with early and late onset of HDPs (diagnosed at < 35 weeks vs ≥ 35 weeks). The early and late-onset models included weekly exposures from 12 weeks of preconception up to 34 and 42 weeks, respectively.

We also investigated the non-linear cumulative effects of the exposure during the preconception through pregnancy, preconception, entire pregnancy, and for each trimester with standard logistic regression. Thus, a *one-basis* instead of *cross-basis* function in the “dlnm” R package was used to construct a unidimensional exposure-outcome association using natural splines with the 4 *dfs.* Preconception and entire pregnancy exposures were included simultaneously in one model to minimize bias (Wilson et al. 2017). All models were adjusted for the covariates or potential confounders described earlier. Maternal age was modeled as a continuous variable using natural splines with 3 *dfs* (Hviid et al. 2022; Nyadanu et al. 2023a; Strand et al. 2011a).

Potential effect modification was explored through stratified analyses by sex (male or female), race /ethnicity (Caucasian or non-Caucasian), maternal age (< 35 or ≥ 35 years old), parity (nulliparity or multiparity), SES (high, moderate, low), and smoking during pregnancy (smoker or non-smoker). The cumulative UTCI exposures over 12 weeks of preconception through pregnancy at the exposure threshold that showed consistent positive associations with reference to the median were reported for the subgroups.

The following sensitivity analyses were performed to test the stability of the main results: (i) mean rather than median UTCI was used as a reference, (ii) maternal age was included as a categorical variable (≤ 19, 20–34, ≥ 35) instead of natural splines of a continuous variable, (iii) season of conception was adjusted as four-season categories (autumn, winter, spring, summer) instead of calendar month.

(iv) preconception period was excluded to examine from conception through pregnancy, (v) The *dfs* in the natural cubic spline in the cross-basis function of the DLNM were changed to 5 and 4 *dfs* for exposure–response and exposure period-response associations, respectively and (vi) trimester-specific effects were also estimated by including three trimester-specific average exposures in one model simultaneously to minimize bias in effect estimates (Neophytou et al. 2021; Wilson et al. 2017) as well as analyzing three separate trimester-specific average exposures in individual models.

## Results

### Study population and bioclimatic exposure

A total of 415,091 singleton pregnancies or births were included, of which 15342 (3.7%) and 11456 (2.8%) were clinically diagnosed as gestational hypertension and preeclampsia, respectively. Of these, 1157 (0.3%) and 14185 (3.4%) were early and late-onset gestational hypertension, respectively, and 2662 (0.6%) and 8794 (2.1%) were early and late-onset preeclampsia, respectively. There were slightly more male (51.2%) than female births (48.8%). Most of the pregnant women were Caucasians (78.3%), married (87.3%), non-smokers (85.3%), multiparous (58.1%), and lived in cities (61.9%). Season of conception was almost uniformly distributed (Table 1). The range of the average bioclimatic index (UTCI) exposure for preconception through pregnancy was 6.9 to 32.1 °C with a mean of 14.5 ± 2.5 °C (standard deviation) and median of 14.2 °C which were almost identical to that of the specific average exposures for preconception, pregnancy, and each trimester (Table 2). The exposure varied within each specific pregnancy week ranging from − 11.0 °C in the 41st gestational week to 39.1 °C in the 36th gestational week.

**Table 1:** Maternal characteristics of included singleton pregnancies in Western Australia, 2000–2015 (*N* = 415,091)

Characteristics	Category	*n* (%)
Gestational hypertension	No	399,749 (96.3)
	Yes	15,342 (3.7)
	- Early-onset	1,157 (0.3)
	- Late-onset	14,185 (3.4)
	No	403,635 (97.2)
	Yes	11,456 (2.8)
Preeclampsia	- Early-onset	2,662 (0.6)
	- Late-onset	8,794 (2.1)
Sex of the baby	Male	212,513 (51.2)
	Female	202,578 (48.8)
Race/ethnicity	Caucasian	325,084 (78.3)
	Non-Caucasian	90,007 (21.7)
Marital status	Married	362,365 (87.3)
	Unmarried	52,726 (12.7)
Smoked during pregnancy	No	354,079 (85.3)
	Yes	60,989 (14.7)
	Unknown	23 (0.0)
Parity	Nulliparity	173,881 (41.9)
	Multiparity	241,210 (58.1)
Remoteness	Urban	257,106 (61.9)
	Rural	157,846 (38.0)
	Unknown	139 (0.0)
SES	High	138,357 (33.3)
	Moderate	138,356 (33.3)
	Low	138,356 (33.3)
	Unknown	22 (0.0)
Maternal age (years)	20–34	312,902 (75.4)
	≤ 19	19,015 (4.6)
	≥ 35	83,174 (20.0)
Season of conception	Autumn	101,026 (24.3)
	Winter	104,991 (25.3)
	Spring	104,973 (25.3)
	Summer	104,101 (25.1)

*SES* socioeconomic status

**Table 2:** Descriptive statistics of the weekly average UTCI (℃) from 12 weeks preconception through to the gestational week at diagnosis of gestational hypertension or preeclampsia or birth delivery for the study cohort of singleton pregnancies in Western Australia, 2000–2015 (*N* = 415,091)

Exposure periods	Min	Mean ± SD	Median	P1	P5	IQR	P95	P99	Max
Preconception through pregnancy	6.9	14.5 ± 2.5	14.2	10.2	11.9	1.2	17.4	26.0	32.1
Preconception	1.4	14.4 ± 5.2	14.0	5.8	7.6	8.8	22.0	29.5	35.8
Pregnancy	4.8	14.6 ± 2.9	14.2	9.6	11.2	2.9	18.4	26.8	34.1
1st trimester	1.7	14.6 ± 5.2	14.2	5.9	7.7	8.8	22.0	29.6	36.0
2nd trimester	0.0	14.6 ± 5.2	14.2	6.1	7.8	8.7	22.0	29.8	36.1
3rd Trimester	− 1.0	14.4 ± 5.3	14.0	5.2	7.6	8.8	22.0	29.7	36.0

*UTCI* Universal Thermal Climate Index, *SD* standard deviation, *P1-P99* 1st to 99th centiles, *IQR* interquartile range = P75-P25

### Bioclimatic exposure and the odds of HDPs

**Fig. 1:**
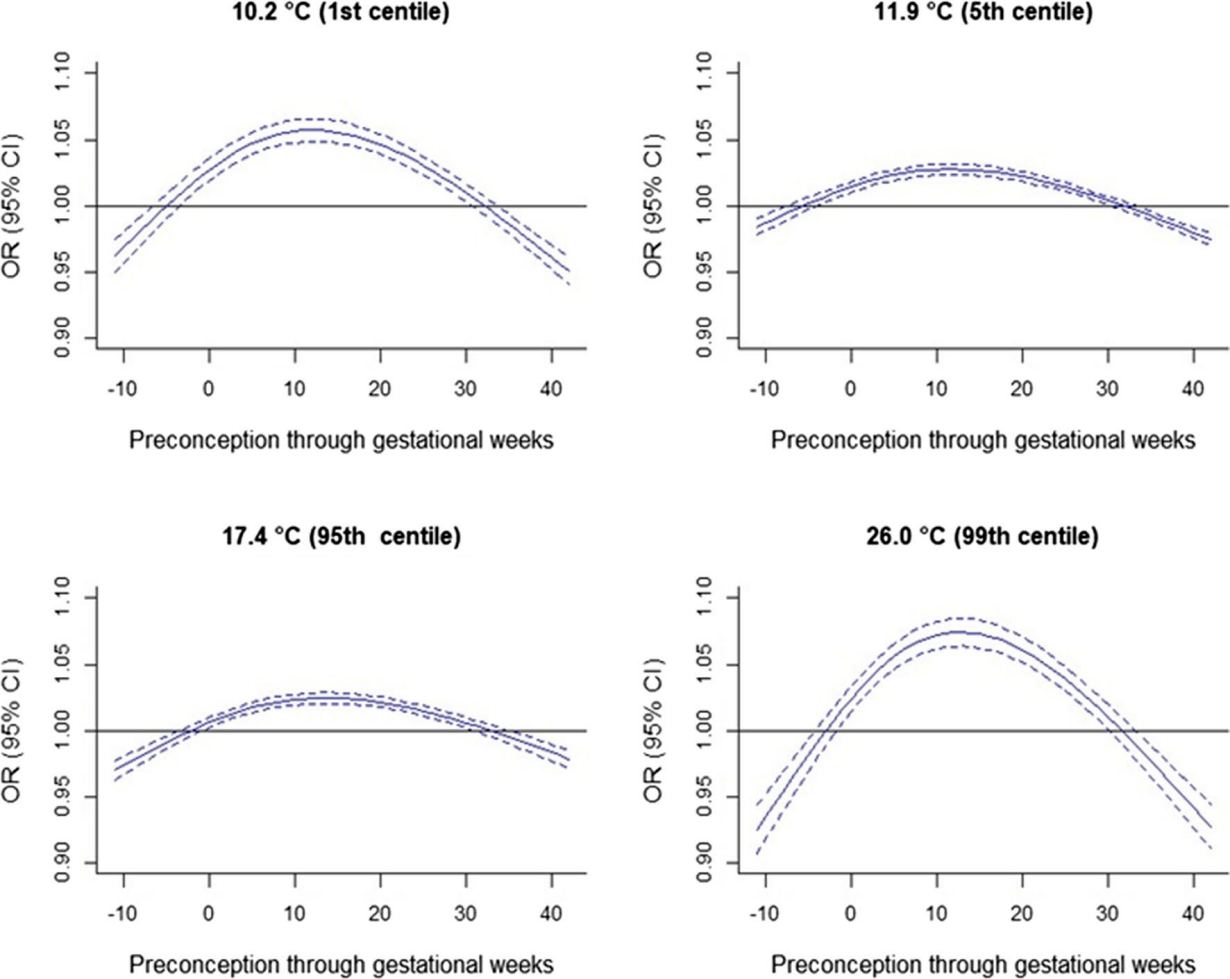
Weekly-specific UTCI for 12 weeks preconception (− 11 to 0) through to gestational week (1 to 42) and the odds of gestational hypertension at different thresholds of UTCI with reference to the median of 14.2 ◦C. The blue solid lines represent point estimates, and the broken lines represent 95% confidence intervals. All models were adjusted for infant sex, maternal age, race or ethnicity, marital status, smoking status, parity, remoteness, socioeconomic status, and year and month of conception. Note: OR, odds ratio; CI, confidential interval; UTCI, Universal Thermal Climate Index

**Fig. 2:**
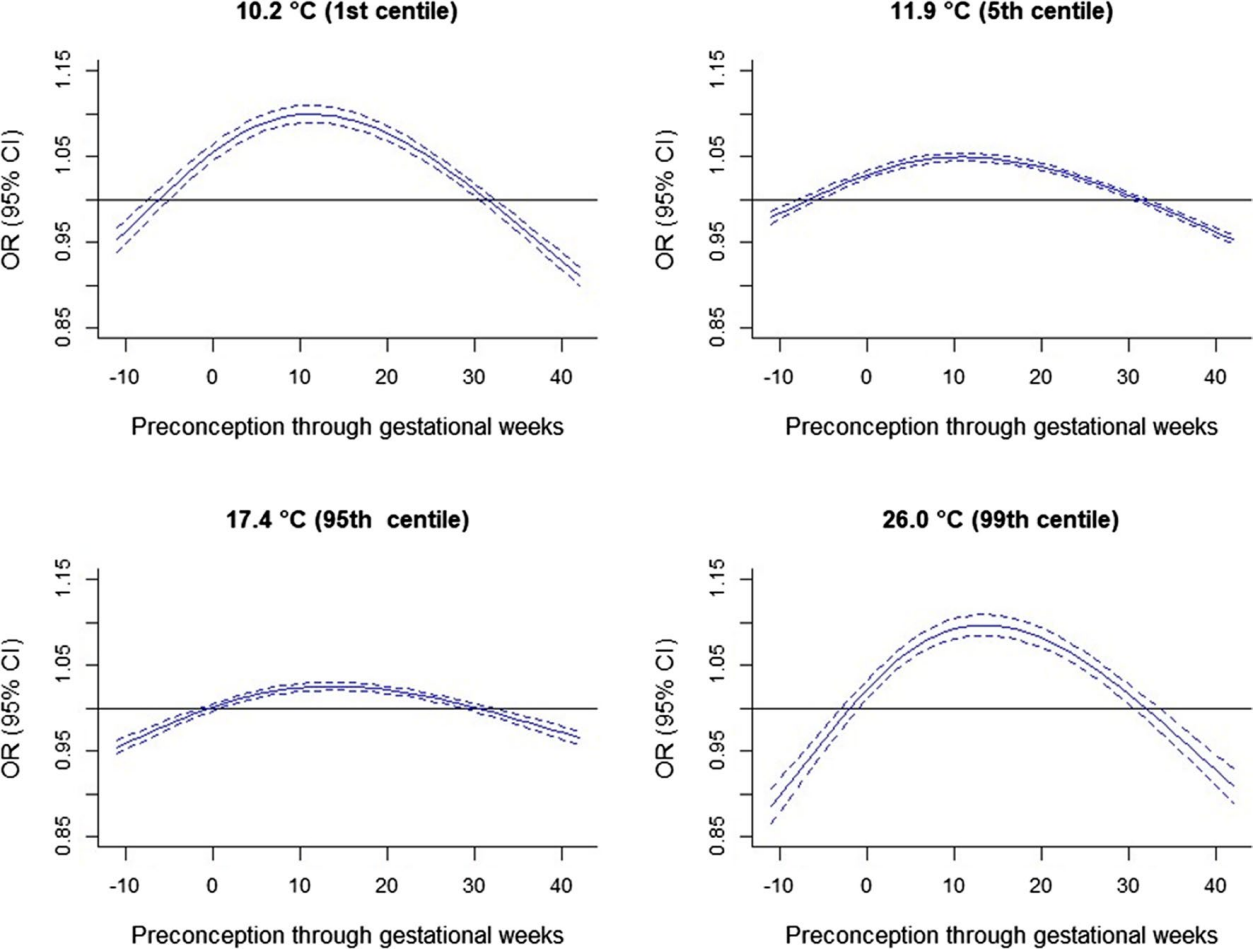
Weekly-specific UTCI for 12 weeks preconception (-11 to 0) through to gestational week (1 to 42) and the odds of preeclampsia at different thresholds of UTCI with reference to the median of 14.2 ◦C. The blue solid lines represent point estimates, and the broken lines represent 95% confidence intervals. All models were adjusted for infant sex, maternal age, race or ethnicity, marital status, smoking status, parity, remoteness, socioeconomic status, and year and month of conception. Note: OR, odds ratio; CI, confidential interval; UTCI, Universal Thermal Climate Index

Exposure to different centiles of weekly UTCI as compared to median exposure showed inverted “U”-shaped associations with both HDPs. Exposure in preconception weeks and after 30th gestational weeks showed lower odds of HDPs. Exposure from early pregnancy up to 30th gestational weeks is associated with greater odds of HDPs with critical periods of vulnerability at all exposure thresholds, particularly elevated at the 1st (10.2 °C) and 99th (26.0 °C) exposure centiles as compared to the median (14.2 °C). Exposure at the 99th centile as compared to the median showed the most elevated weekly ORs of 1.07 (95% CI 1.06, 1.08) in gestational weeks 8–18 for gestational hypertension (Fig. 1, Table S1). The most elevated ORs of preeclampsia were 1.10 (95% CI 1.08, 1.11) at 99th centile in gestational weeks 11–16 and 1.10 (95% CI 1.09, 1.11) at 1st exposure centile in gestational weeks 8–15 (Fig. 2, Table S2). The unadjusted exposure-lag-response curves were almost identical to the adjusted models (Figure S2).

Results of the early and late onsets of HDPs differed slightly from the overall HDPs. As compared to the median exposure, lower exposures (1st and 5th UTCI centiles) showed nearly linear associations with early-onset gestational hypertension which increased towards the end of gestation. Higher exposures (95th and 99th UTCI centiles) showed U-shaped associations with early-onset gestational hypertension. However, late-onset gestational hypertension indicated similar patterns found in the association of the exposure to overall gestational hypertension (Figure S3). Early-onset preeclampsia at all exposure centiles, but stronger at lower centiles as compared to median exposure showed nearly linear and increasing odds toward the end of gestation. The association of UTCI with late-onset preeclampsia had an inverted U-shape as in the overall preeclampsia (Figure S4).

Cumulative exposure from preconception through pregnancy only showed higher odds of gestational hypertension at the 95th UTCI centile as compared to median exposure, 1.34 (95% CI 1.23, 1.45) while exposure at other centiles showed relatively lower odds of gestational hypertension. Greater odds of preeclampsia were found at all exposure thresholds and most elevated at the 95th UTCI centile as compared to the median for preconception through pregnancy 1.74 (95% CI 1.59, 1.91). For specific exposure periods, the most elevated ORs in preconception and pregnancy periods were 1.09 (95% CI 0.98, 1.22) and 1.89 (95% CI 1.74, 2.06) at 95th UTCI centile for gestational hypertension, respectively, and 1.64 (95% CI 1.40, 1.91) and 3.07 (95% CI 2.75, 3.42) at the 1st UTCI centile for preeclampsia, respectively (Table 3).

**Table 3:** The associations between maternal cumulative UTCI exposures from 12 weeks preconception through pregnancy, and odds ratios of gestational hypertension and preeclampsia at various exposure thresholds with reference to median exposure of 14.2 ˚C for the study cohort

Exposure period		Gestational hypertension	Preeclampsia
	UTCI centile	OR (95% CI)	OR (95% CI)
Preconception through pregnancy	P1	0.80 (0.72, 0.88)	1.61 (1.45, 1.79)
	P5	0.80 (0.75, 0.86)	1.57 (1.46, 1.69)
	P95	1.34 (1.23, 1.45)	1.74 (1.59, 1.91)
	P99	0.86 (0.77, 0.95)	1.17 (1.05, 1.32)
Preconception	P1	0.85 (0.74, 0.98)	1.64 (1.40, 1.91)
	P5	0.86 (0.76, 0.96)	1.29 (1.13, 1.46)
	P95	1.09 (0.98, 1.22)	1.13 (1.01, 1.27)
	P99	0.82 (0.68, 1.00)	0.81 (0.66, 1.00)
Pregnancy	P1	1.27 (1.14, 1.41)	3.07 (2.75, 3.42)
	P5	1.15 (1.07, 1.24)	2.16 (1.99, 2.34)
	P95	1.89 (1.74, 2.06)	2.54 (2.32, 2.79)
	P99	0.97 (0.81, 1.17)	1.67 (1.37, 2.04)

Standard non-linear logistic regression model adjusted for infant sex, maternal age, race, marital status, parity, maternal smoking, remoteness, areal level socioeconomic status, year, and calendar month of conception. *P1-P99* first to 99th centile of UTCI, *UTCI* Universal Thermal Climate Index in degree Celsius, *OR* odds ratio, *CI* confidence intervals

The exposure at the 95th UTCI centile as compared to the median which showed consistent positive associations with both HDPs over the preconception through pregnancy periods maintained the positive associations in all subgroups with clearly greater magnitude in some subgroups. Within each group, the ORs were more elevated in males for gestational hypertension, 1.40 (95% CI 1.25, 1.57) but in females for preeclampsia, 1.81 (95% CI 1.59, 2.06). Caucasians were at greater odds for both gestational hypertension, 1.49 (95% CI 1.35, 1.64), and preeclampsia 1.94 (95% CI 1.73, 2.17). Non-smokers were at greater odds of HDPs for gestational hypertension, 1.41 (95% CI 1.29, 1.55), and preeclampsia, 1.84 (95% CI 1.67, 2.04). Birth to mothers aged < 35 years old were at greater odds of gestational hypertension, 1.36 (95% CI 1.24, 1.48) but those ≥ 35 years old were at greater odds of preeclampsia, 2.54 (95% CI 2.03, 3.17). Multiparous mothers were at greater odds for both gestational hypertension, 1.47 (95% CI 1.31, 1.65), and preeclampsia, 1.90 (95% CI 1.65, 2.18). Residing in moderate SES showed greater odds for both gestational hypertension, 2.09 (95% CI 1.69, 2.58), and preeclampsia, 2.85 (95% CI 2.27, 3.59) (Table 4).

**Table 4:** The associations between maternal cumulative UTCI exposure 12 weeks preconception through pregnancy and odds ratios of gestational hypertension and preeclampsia at the 95th centile of exposure with reference to median exposure of 14.2 ˚C for the study cohort

Variables	Group variables	Gestational hypertension	Preeclampsia
		OR (95% CI)	OR (95% CI)
Sex of the baby	Male	1.40 (1.25, 1.57)	1.70 (1.50, 1.92)
	Female	1.28 (1.14, 1.44)	1.81 (1.59, 2.06)
Race/ethnicity	Caucasian	1.49 (1.35, 1.64)	1.94 (1.73, 2.17)
	Non-Caucasian	1.08 (0.92, 1.27)	1.53 (1.32, 1.77)
Smoking status	Smoker	1.05 (0.86, 1.28)	1.45 (1.19, 1.78)
	Non-smoker	1.41 (1.29, 1.55)	1.84 (1.67, 2.04)
Maternal age	≥ 35 years old	1.27 (1.03, 1.57)	2.54 (2.03, 3.17)
	< 35 years old	1.36 (1.24, 1.48)	1.64 (1.49, 1.81)
Parity	Nulliparity	1.23 (1.09, 1.38)	1.67 (1.48, 1.88)
	Multiparity	1.47 (1.31, 1.65)	1.90 (1.65, 2.18)
SES	High SES	1.17 (1.04, 1.32)	1.61 (1.42, 1.83)
	Moderate SES	2.09 (1.69, 2.58)	2.85 (2.27, 3.59)
	Low SES	1.43 (1.17, 1.74)	2.67 (2.13, 3.34)

*SES* socioeconomic status, *OR* odds ratios, *CI* confidence interval, *UTCI* Universal Thermal Climate Index

The results of all the sensitivity analyses described earlier were generally consistent with those of the main results (Figure S5–S9), confirming the stability of the results under the variations in the model assumptions. All three trimester-average exposures generally showed positive associations with both HDPs. The most elevated odds of gestational hypertension were at the 95th UTCI centile in the first trimester but at the 99th exposure centile in the third trimester from the simultaneous and separate models, respectively. However, the odds of preeclampsia were consistently most elevated at 1st UTCI centile in the third trimester from both simultaneous and separate trimester-average exposure models (Table S3).

## Discussions

### Main findings and interpretations

Weekly maternal exposure to UTCI showed increased odds at both low (1st and 5th centiles) and high (95th and 99th centiles) UTCI exposures as compared to the median (14.2 °C) with critical windows of susceptibility demonstrated by an inverted “U”-shaped pattern of associations with the HDPs over the period from preconception through pregnancy. Lower odds of HDPs associated with low and high UTCI centiles were also found in preconception weeks and after the 30th gestational week. Weekly exposures from early to the 30th gestational weeks increased the odds of HDPs at all exposure centiles compared to the median, with particularly elevated odds at the 1st (10.2 °C) and 99th (26.0 °C) UTCI centiles. We found the greatest odds of HDPs at the 99th centile exposure, especially in gestational weeks 8–18 for gestational hypertension and the same magnitude during weeks 11–16 at the 99th centile and weeks 8–15 at the 1st centile for preeclampsia. The exposure was associated somewhat linearly with early-onset HDPs but the late-onset HDPs showed almost similar patterns as that of the overall HDPs. The effects of the exposure were greater in early than late-onset HDPs. While higher odds of preeclampsia were found at all exposure centiles for cumulative preconception through pregnancy exposures, greater odds of gestational hypertension were found at only the 95th exposure centile with lower odds at most exposure centiles. The greatest odds were found at the 95th and 1st exposure centiles for gestational hypertension and preeclampsia, respectively. Trimester-average exposures showed the greatest odds of gestational hypertension in the first trimester at 95th centile and preeclampsia in the third trimester at 1st exposure centile. Exposure at the 95th UTCI centile consistently showed positive associations with both HDPs in all subgroups, and the associations were stronger in certain subgroups. For all exposure periods and subgroups examined, the odds of preeclampsia, including early or late onset were stronger than that of gestational hypertension.

To the best of our knowledge, this is the first study to investigate critical periods for the effects of bioclimatic stress (UTCI) exposure on HDPs. Few studies investigated the long-term effects of ambient temperature with weekly-specific exposure (Part et al. 2022; Qian et al. 2023; Youssim et al. 2023; Zeng et al. 2024). Our weekly preconception did not but cumulative 12 weeks preconception showed a positive association, particularly for preeclampsia. Although with higher effect estimates, our findings were somewhat consistent with a previous finding that reported greater odds of HDPs, especially for preeclampsia for cumulative 12 weeks preconception average temperature below the 10th, but lower odds for temperature exposures above 90th centile (Xiong et al. 2020). That study also found lower odds of HDPs for average temperature exposures below the 10th centile and greater odds of HDPs for average temperature exposures above the 90th centile during the first half of pregnancy (1–20 weeks) (Xiong et al. 2020). Another study found no association between temperature exposure during the cumulative 12 preconception weeks and the risk of preeclampsia (Youssim et al. 2023). However, our results for preeclampsia showed greater odds for both low and high UTCI exposure during the cumulative 12 weeks of preconception periods while only 95th UTCI centile showed greater odds of gestational hypertension.

Of the four studies that investigated weekly ambient temperature and HDPs, one study found that weekly ambient temperature from conception up to the 20th gestational week (date of diagnosis) at both low (2nd, 5th, and 10th centiles) and high (90th, 95th, 98th centiles) temperatures relative to the median temperature associated with lower odds of gestational hypertension with low temperatures showing critical protective periods in the 4th–5th and 14th–16th gestational weeks in Beijing, China. The other three studies, however, in Johannesburg, South Africa (Part et al. 2022), the Southern District of Israel (Youssim et al. 2023), and Guangzhou, a megacity in Southern China (Zeng et al. 2024) found positive associations with critical susceptible periods. In the first study, the authors assessed two mutually exclusive composite outcomes as high blood pressure, hypertension, or gestational hypertension (hBP) and preeclampsia, eclampsia or severe pre-eclampsia or imminent eclampsia, or HELLP (hemolysis, elevated liver enzymes, and low platelets) syndrome (PEH) (Part et al. 2022). As compared to the median temperature of 18 ˚C, they found that exposure to high mean weekly temperatures (95th centile, 23 °C) in early pregnancy (2–5 weeks) and low mean weekly temperature (5th centile, 11 °C) between 7 and 34th gestational weeks were associated with greater hazards of PEH. High temperatures during the first few weeks of pregnancy showed a tendency for greater hazards of hBP and low temperatures during the 29th week onwards (third trimester) associated with greater hazards of hBP. The findings were fairly consistent with our results in terms of the directions of association but with far greater magnitude of the effect estimates than ours and different critical susceptible periods most likely due to the different exposure metrics and outcomes assessments. For example, as compared to median exposure, the most elevated hazards were 1.79 (95% CI 1.19, 2.71) at the 95th exposure centile in the fourth week of pregnancy for PEH and 1.86 (95% CI 1.36, 2.53) at the 5th exposure centile in the 37th gestational week (Part et al. 2022). Our most elevated odds were 1.07 (95% CI 1.06, 1.08) in gestational weeks 8–18 for gestational hypertension at 99th exposure centile and 1.10 (95% CI 1.08, 1.11) at 99th exposure centile in gestational weeks 11–16 or 1.10 (95% CI 1.09, 1.11) at 1st exposure centile in gestational weeks 8–15 for preeclampsia. The second study found that weekly high temperatures at the beginning and, particularly, at the end of gestation, were associated with an increased risk of preeclampsia. Those authors found that during gestational week 33, the cause-specific hazard ratio of preeclampsia was 1.01 (95% CI 1.01, 1.02) at 30 °C exposure, 1.05 (95% CI 1.03, 1.08) at 35 °C, and 1.07 (95% CI 1.04, 1.10) at 37 °C as compared to the median temperature of 22.4 °C (Youssim et al. 2023). The third study also found gestational weeks 1–8 as critical susceptible exposure periods for preeclampsia (Zeng et al. 2024). Generally, the previous studies indicated early and late gestational weeks as critical susceptible periods (Part et al. 2022; Youssim et al. 2023; Zeng et al. 2024), but our findings indicated early to 30th gestational weeks after which there were lower odds of HDPs. This implies that the association between ambient temperature or bioclimatic exposures and the risk of HDPs is more likely due to long-term exposure accumulating from early pregnancy rather than short-term exposure (Zeng et al. 2024). This is because defective placentation is the proposed pathogenesis of early-onset preeclampsia and interactions between normal placental senescence and a maternal genetic predisposition to chronic cardiovascular and metabolic diseases are implicated in late-onset preeclampsia (Burton et al. 2019). The observed lower odds could be due to attitudinal changes to minimize outdoor activities during late pregnancy, especially during extreme bioclimatic exposures. Similarly, the observed comparatively greater odds of HDPs for cumulative exposure at the 95th centile than 99th centile of UTCI could be due to higher tendencies of pregnant women minimizing outdoor activities during extreme exposure (99th UTCI centile) as compared to severe exposure (95th UTCI centile). In addition to the different exposure metrics and outcome assessments as major factors, other factors such as the geography, characteristics of the study population, and acclimatization could explain the differences in the findings.

In a mutually adjusted model, the study from Southern Israel reported positive associations between temperature and preeclampsia for all trimesters, with the most elevated and consistent positive association found in the second trimester at high temperatures (Youssim et al. 2023). We also found a positive association between UTCI and preeclampsia for all trimesters but at both low and high exposure levels with the most elevated positive association found in the third trimester at low exposure level (1st UTCI centile). Moreover, both our results and the most elevated critical period from weekly exposure found in that study did not align with the trimester-specific exposures. This reaffirmed a simulation study’s finding that trimester-average exposures could lead to biased estimates with incorrectly identified critical windows (Wilson et al. 2017). Thus, the application of DLNM to mitigate this bias and to identify shorter critical susceptible periods is highly recommended in future studies (Gasparrini 2014; Wilson et al. 2017). A recent systematic review and meta-analysis included 15 primary studies with five studies included in a meta-analysis (Mao et al. 2023). The authors found that heat and cold exposures had opposite effects on HDPs, indicating bidirectional effects of extreme temperatures during both 12 weeks of preconception (based on a single study) and weeks 1–20 of pregnancy. However, no consistent conclusion was found for the effects of extreme temperatures on HDPs after 20 weeks of gestation, suggesting more comprehensive studies with appropriate designs (Mao et al. 2023). Thus, more studies from other geodemographic populations using bioclimatic exposure metrics such as UTCI (Romaszko et al. 2022; Staiger et al. 2019; Vanos et al. 2020) and applying DLNM methodology (Gasparrini 2014; Wilson et al. 2017) for long-term effects rather than a specific short period of time, including the preconception period (Mao et al. 2023) to examine the thermal-HDP associations to better understand the critical susceptible periods for timely interventions are suggested. Moreover, due to their unique morphophysiology, developing bioclimatic metrics specifically for pregnant women is essential to characterize the true impacts of bioclimatic exposure on pregnancy outcomes.

Together, the existing epidemiological evidence indicates that maternal thermal stress (cold or heat) exposure from preconception to early stages (pre-implantation to early placental development), and late in pregnancy are possible contributing factors to HDPs (Mao et al. 2023; Part et al. 2022). Two to three months before and after conception are critical periods in reproductive health where environmental exposures have the potential to induce epigenetic alterations in the gamete structure and placental development, leading to adverse pregnancy outcomes and long-term adverse health outcomes (Keikha et al. 2022). Thus, preconception to early pregnancy when women may be unaware of pregnancy is a key time to modify, prevent, and manage risk factors to improve pregnancy outcomes and the future health of the offspring (Keikha et al. 2022). The mid to late stages of pregnancy are also associated with high fetal growth and metabolism which increase the thermal vulnerability of pregnant women (Erez et al. 2017). Given that many climate-related health risks are preventable, appropriate climate-related action plans involving behavioral strategies, biophysical solutions, climate-related healthcare systems, and policy are needed (Ebi et al. 2021; Giudice et al. 2021; Nyadanu et al. 2023b, 2022b).

Although high exposure showed positive associations with HDPs in all subgroups, the effect was further elevated in specific vulnerable subgroups, which varied between gestational hypertension and preeclampsia. However, the effect of the exposure was consistently elevated in both HDPs for Caucasians, non-smokers, multiparous, and mothers in moderate SES residences. Multiparous pregnant women are more likely to have other pre-existing obstetrical conditions that put them at greater risk. It was particularly surprising to find that Caucasians were at higher risk, but this could be explained through thermal acclimatization. As compared to Caucasians and most likely in high SES residential areas, non-Caucasians in moderate and low SES areas are more likely to be under-resourced for thermal mitigation strategies such as the use of air conditioning and to engage in occupations that demand prolonged physical work in hot and humid environments (Adnan et al. 2022). The Indigenous people (Aboriginal and Torres Strait Islanders) from regional areas and migrants from tropical and sub-tropical regions may have developed better acclimatization, especially to heat exposure. For instance, a recent study in the Northern Territory of Australia found that Indigenous are not more susceptible to heat mortality than non-Indigenous and that excessive use of air-conditioners might impair physiological acclimatization to the prevailing environment (Quilty et al. 2023). Therefore, physiological and sociocultural adaptations also have relative value as potentially useful mechanisms in preparing for future extreme climate change (Quilty et al. 2023). We also surprisingly observed that the exposure had greater odds of both HDPs in non-smokers than smokers. A recent meta-analysis of 13 studies also found that smoking during pregnancy was associated with lower odds of developing HDPs (Wang et al. 2022). While there is probably no single epidemiological explanation for this effect of smoking on HDP risks, this puzzling finding could be due to several sources of bias. These include, but are not limited to eligibility criteria, early losses of at-risk pregnancies, competing events (e.g., preterm birth, stillbirth, placental abruption) which may prevent the incidence of HDPs by ending the pregnancy, the definition and misclassification of HDPs, measurement errors of smoking, inadequate adjustment, and unmeasured confounders (Rodriguez-Lopez et al. 2023). Like our study, some other key covariates or confounders of HDPs such as alcohol and illicit drug use, maternal body mass index (BMI), and psychological stress were not adjusted for in the previous studies reviewed elsewhere (Mao et al. 2023) as these variables are hardly measured in routine population-based cohorts. A recent study included BMI and found that women with higher pre-pregnancy BMI were more susceptible to high temperatures and longer critical exposure periods for preeclampsia than those with normal or lower BMI (Zeng et al. 2024). But it is worth noting that the pre-pregnancy BMI in that study was an effect modifier, not a confounder. However, a high-quality longitudinal cohort with sufficient covariates will improve the effect estimates and critical susceptible periods identification.

### Biological mechanisms

The pathogenesis of pre-eclampsia is characterized by aberrant spiral artery remodeling, placental ischemia, oxidative stress at the maternal–fetal interface, and angiogenic imbalance in the mother’s circulation, which causes endothelial and end-organ damage (Phipps et al. 2019). Thermal or biothermal stress (heat or cold stress) also causes oxidative stress and oxidative generation of excess reactive oxygen species, decreases uterine and placental blood flow, elevates proinflammatory cytokines levels, and disrupts endocrine activities (Collier et al. 2017; Edwards et al. 2003; Lian et al. 2017, 2020; Ziskin and Morrissey 2011; Nyadanu et al. 2024). These affect biological processes such as placental physiology, blastocyst implantation and placentation, and maternal–fetal tolerance where the endpoint is the pregnancy complications (Ayinzat et al. 2021; Berestoviy et al. 2021; Edwards et al. 2003; Lian et al. 2020; Saghafi et al. 2018; Tamási et al. 2010). Low levels of proangiogenic factors and high levels of antiangiogenic factors which served as theranostic agents in clinical trials are key biomarkers for early diagnosis and prognosis of preeclampsia (Phipps et al. 2019). Also, hyperthermia has an anti-angiogenic action (Roca and Primo 2006; Sawaji et al. 2002), suggesting the causal plausibility of thermal stress on HDPs through the elevation of anti-angiogenic factors (Phipps et al. 2019).

### Strengths and limitations

This study has several advantages. First, this study benefited from the use of advanced Fiala-UTCI, which has been demonstrated as a very suitable bioclimatic metric (UTCI) (Blazejczyk et al. 2013; Bröde et al. 2012; Fiala et al. 2012; Jendritzky et al. 2012; Staiger et al. 2019) for medical and epidemiologic studies, and thermal-health warning systems in the context of climate change (Krüger 2021; Romaszko et al. 2022). Second, UTCI exposure was assessed spatiotemporally (Di Napoli et al. 2021) to minimize exposure misclassification as compared to the use of simple models or proximity to sparse monitoring sites. Third, we used DLNM to account for both delayed effect and intensity of the exposure (Gasparrini 2014; Gasparrini et al. 2010) from preconception through pregnancy (Keikha et al. 2022) to investigate unbiased weekly-specific dose–response associations (Gasparrini 2014; Gasparrini et al. 2010; Wilson et al. 2017) in addition to cumulative average exposure over larger windows such as preconception, pregnancy, and trimester-specific periods. Fourth, the large sample size, diagnosis during the entire pregnancy period which included diagnosis before 20 gestational weeks, and access to the date of diagnosis allowed us to further investigate early and late-onset HDPs. Fifth, this is the first study on the topic in Australia.

We also acknowledge the limitations of our study. First, we did not have data on indoor air conditioning usage, time spent indoors or outdoors, maternal activity-time patterns, and residential mobility which could influence exposure assessment (Heo et al. 2023) and bias our effect estimates toward the null. These issues and residual confounding could be addressed through individual-level prospective cohort design with a detailed collection of the essential variables and using mobile thermal sensors for personalized real-time-activity exposure measurements. However, due to time and resource constraints, this method is inherently not feasible for large-scale studies to ensure sufficient power for detecting the effect estimate. Second, despite the availability of the date of diagnosis, the exact onset of HDPs is unknown and women could develop the disease before clinical diagnosis at admission. Third, as in all observational studies, residual confounding is an inherent limitation. We did not have data on certain risk factors for HDPs such as maternal lifestyle (e.g., alcohol or illicit drug intake, physical activity), psychological stress, nutritional status, infection, maternal weight, and height for the whole study period. However, most of these covariates would not be associated with UTCI or other climatic variables and it is unlikely that their omission would confound our results, particularly given that our unadjusted and adjusted exposure-lag-response curves were almost identical. Moreover, adjustment for SES and remoteness provided secondary control for some of these covariates. Improving the quality of data collection in the routine population-based cohorts could help minimize the issues of residual confounding. Fourth, bioclimatic metrics for the general population may underestimate the effects so future bioclimatic metrics that integrate the unique morphophysiological characteristics of pregnant women will make effect estimates more reliable and realistic.

## Conclusions

This study reveals that maternal exposure to both low and high bioclimatic exposure as compared to median exposure from preconception through pregnancy was associated with hypertensive disorders of pregnancy with indication of critical susceptible periods (weeks 8–18). The exposure–response associations were greater in early than late onset of hypertensive disorders of pregnancy. Despite positive associations in all subgroups, specific subgroups were more vulnerable, which varied between gestational hypertension and preeclampsia. Across all exposure periods, early or late onsets, and subgroups examined, the odds of preeclampsia were stronger than those of gestational hypertension. Given increasing climate change concerns, we suggest the consideration of climate-related factors in managing reproductive health outcomes. Clinicians should counsel women at reproductive age on environmental exposures, especially during early to mid-pregnancy periods to promote healthy pregnancy and healthy offspring. Thermal health warning systems and further studies using a bioclimatic index are required.

## Supplementary Information

Below is the link to the electronic supplementary material.

Supplementary file1 (DOCX 4188 KB)

## Data Availability

The Universal Thermal Climate Index (UTCI) data is provided by Copernicus Climate Data Store as open access (https://doi.org/10.24381/cds.553b7518). The health data can be requested directly from the Department of Health, Western Australia (https://ww2.health.wa.gov.au/Articles/J_M/Midwives-Notification- System) as it cannot be made available publicly due to the data access agreement.
