# Supplementary material for: Maternal exposure to bioclimatic stress and hypertensive disorders of pregnancy in Western Australia: identifying potential critical windows of susceptibility
Source: Environ Sci Pollut Res Int. 2024 Aug 15;31(39):52279–92. doi: 10.1007/s11356-024-34689-6 (PMC11374825; doi:10.1007/s11356-024-34689-6)
Supplement: Supplementary file 1 — Supplementary file1 (DOCX 4188 KB) [file 11356_2024_34689_MOESM1_ESM.docx]

**Maternal exposure to bioclimatic stress and hypertensive disorders of pregnancy in Western Australia: identifying potential critical windows of susceptibility**

Amanuel T. Gebremedhin,^1¥^ Sylvester D. Nyadanu,^1,2 ¥*^ Ivan C. Hanigan^1,3^, Gavin Pereira^1,3,4^

^1^Curtin School of Population Health, Curtin University, Perth, Kent Street, Bentley, Western Australia 6102, Australia

^2^Education, Culture, and Health Opportunities (ECHO) Ghana, ECHO Research Group International, Aflao, Ghana

^3^WHO Collaborating Centre for Climate Change and Health Impact Assessment. Faculty of Health Science, Curtin University, WA, Australia

^4^enAble Institute, Curtin University, Perth, Kent Street, Bentley, Western Australia 6102, Australia

¥ Co-first authors

*Address correspondence to

Curtin School of Population Health, Curtin University, Perth, Kent St., Bentley, Western Australia 6102, Australia.

Email: [sylvester.nyadanu@curtin.edu.au](mailto:sylvester.nyadanu@curtin.edu.au) (Nyadanu SD)

Table S1. The associations between maternal mean weekly UTCI exposures over twelve weeks preconception through to pregnancy and the odds of gestational hypertension at various exposure thresholds with reference to median 14.2 ˚C in Western Australia, 2000–2015.

| Week | P1 (10.2 ˚C) | | | P5 (11.9 ˚C) | | | P95 (17.5 ˚C) | | | P99 (26.1 ˚C) | | |
| --- | --- | --- | --- | --- | --- | --- | --- | --- | --- | --- | --- | --- |
|  | OR | LCI | UCI | OR | LCI | UCI | OR | LCI | UCI | OR | LCI | UCI |
| -11 | 0.96 | 0.95 | 0.98 | 0.98 | 0.98 | 0.99 | 0.97 | 0.96 | 0.98 | 0.93 | 0.91 | 0.94 |
| -10 | 0.97 | 0.96 | 0.98 | 0.99 | 0.98 | 0.99 | 0.97 | 0.97 | 0.98 | 0.94 | 0.92 | 0.95 |
| -9 | 0.98 | 0.96 | 0.99 | 0.99 | 0.99 | 1.00 | 0.98 | 0.97 | 0.98 | 0.94 | 0.93 | 0.96 |
| -8 | 0.98 | 0.97 | 0.99 | 0.99 | 0.99 | 1.00 | 0.98 | 0.98 | 0.99 | 0.95 | 0.94 | 0.97 |
| -7 | 0.99 | 0.98 | 1.00 | 1.00 | 0.99 | 1.00 | 0.98 | 0.98 | 0.99 | 0.96 | 0.95 | 0.98 |
| -6 | 0.99 | 0.98 | 1.00 | 1.00 | 0.99 | 1.00 | 0.99 | 0.98 | 0.99 | 0.97 | 0.96 | 0.99 |
| -5 | 1.00 | 0.99 | 1.01 | 1.00 | 1.00 | 1.01 | 0.99 | 0.99 | 1.00 | 0.98 | 0.97 | 0.99 |
| -4 | 1.01 | 1.00 | 1.02 | 1.01 | 1.00 | 1.01 | 0.99 | 0.99 | 1.00 | 0.99 | 0.98 | 1.00 |
| -3 | 1.01 | 1.00 | 1.02 | 1.01 | 1.00 | 1.01 | 1.00 | 0.99 | 1.00 | 1.00 | 0.99 | 1.01 |
| -2 | 1.02 | 1.01 | 1.03 | 1.01 | 1.01 | 1.01 | 1.00 | 1.00 | 1.00 | 1.01 | 1.00 | 1.02 |
| -1 | 1.02 | 1.01 | 1.03 | 1.01 | 1.01 | 1.02 | 1.00 | 1.00 | 1.01 | 1.02 | 1.01 | 1.03 |
| 0 | 1.03 | 1.02 | 1.04 | 1.01 | 1.01 | 1.02 | 1.01 | 1.00 | 1.01 | 1.02 | 1.02 | 1.03 |
| 1 | 1.03 | 1.02 | 1.04 | 1.02 | 1.01 | 1.02 | 1.01 | 1.01 | 1.01 | 1.03 | 1.02 | 1.04 |
| 2 | 1.04 | 1.03 | 1.05 | 1.02 | 1.02 | 1.02 | 1.01 | 1.01 | 1.02 | 1.04 | 1.03 | 1.05 |
| 3 | 1.04 | 1.03 | 1.05 | 1.02 | 1.02 | 1.02 | 1.01 | 1.01 | 1.02 | 1.05 | 1.04 | 1.05 |
| 4 | 1.04 | 1.04 | 1.05 | 1.02 | 1.02 | 1.03 | 1.02 | 1.01 | 1.02 | 1.05 | 1.04 | 1.06 |
| 5 | 1.05 | 1.04 | 1.06 | 1.02 | 1.02 | 1.03 | 1.02 | 1.01 | 1.02 | 1.06 | 1.05 | 1.07 |
| 6 | 1.05 | 1.04 | 1.06 | 1.03 | 1.02 | 1.03 | 1.02 | 1.02 | 1.02 | 1.06 | 1.05 | 1.07 |
| 7 | 1.05 | 1.04 | 1.06 | 1.03 | 1.02 | 1.03 | 1.02 | 1.02 | 1.02 | 1.06 | 1.06 | 1.07 |
| 8 | 1.05 | 1.05 | 1.06 | 1.03 | 1.02 | 1.03 | 1.02 | 1.02 | 1.03 | 1.07 | 1.06 | 1.08 |
| 9 | 1.06 | 1.05 | 1.06 | 1.03 | 1.02 | 1.03 | 1.02 | 1.02 | 1.03 | 1.07 | 1.06 | 1.08 |
| 10 | 1.06 | 1.05 | 1.07 | 1.03 | 1.02 | 1.03 | 1.02 | 1.02 | 1.03 | 1.07 | 1.06 | 1.08 |
| 11 | 1.06 | 1.05 | 1.07 | 1.03 | 1.02 | 1.03 | 1.02 | 1.02 | 1.03 | 1.07 | 1.06 | 1.08 |
| 12 | 1.06 | 1.05 | 1.07 | 1.03 | 1.02 | 1.03 | 1.02 | 1.02 | 1.03 | 1.07 | 1.06 | 1.08 |
| 13 | 1.06 | 1.05 | 1.07 | 1.03 | 1.02 | 1.03 | 1.03 | 1.02 | 1.03 | 1.07 | 1.06 | 1.09 |
| 14 | 1.06 | 1.05 | 1.07 | 1.03 | 1.02 | 1.03 | 1.03 | 1.02 | 1.03 | 1.07 | 1.06 | 1.08 |
| 15 | 1.06 | 1.05 | 1.06 | 1.03 | 1.02 | 1.03 | 1.03 | 1.02 | 1.03 | 1.07 | 1.06 | 1.08 |
| 16 | 1.05 | 1.05 | 1.06 | 1.03 | 1.02 | 1.03 | 1.02 | 1.02 | 1.03 | 1.07 | 1.06 | 1.08 |
| 17 | 1.05 | 1.05 | 1.06 | 1.03 | 1.02 | 1.03 | 1.02 | 1.02 | 1.03 | 1.07 | 1.06 | 1.08 |
| 18 | 1.05 | 1.04 | 1.06 | 1.02 | 1.02 | 1.03 | 1.02 | 1.02 | 1.03 | 1.07 | 1.06 | 1.08 |
| 19 | 1.05 | 1.04 | 1.06 | 1.02 | 1.02 | 1.03 | 1.02 | 1.02 | 1.03 | 1.06 | 1.05 | 1.07 |
| 20 | 1.05 | 1.04 | 1.05 | 1.02 | 1.02 | 1.03 | 1.02 | 1.02 | 1.03 | 1.06 | 1.05 | 1.07 |
| 21 | 1.04 | 1.04 | 1.05 | 1.02 | 1.02 | 1.02 | 1.02 | 1.02 | 1.02 | 1.06 | 1.05 | 1.07 |
| 22 | 1.04 | 1.03 | 1.05 | 1.02 | 1.02 | 1.02 | 1.02 | 1.02 | 1.02 | 1.05 | 1.04 | 1.06 |
| 23 | 1.04 | 1.03 | 1.05 | 1.02 | 1.01 | 1.02 | 1.02 | 1.01 | 1.02 | 1.05 | 1.04 | 1.06 |
| 24 | 1.03 | 1.03 | 1.04 | 1.02 | 1.01 | 1.02 | 1.02 | 1.01 | 1.02 | 1.04 | 1.04 | 1.05 |
| 25 | 1.03 | 1.02 | 1.04 | 1.01 | 1.01 | 1.02 | 1.02 | 1.01 | 1.02 | 1.04 | 1.03 | 1.05 |
| 26 | 1.03 | 1.02 | 1.03 | 1.01 | 1.01 | 1.02 | 1.01 | 1.01 | 1.02 | 1.03 | 1.03 | 1.04 |
| 27 | 1.02 | 1.02 | 1.03 | 1.01 | 1.01 | 1.01 | 1.01 | 1.01 | 1.02 | 1.03 | 1.02 | 1.04 |
| 28 | 1.02 | 1.01 | 1.03 | 1.01 | 1.01 | 1.01 | 1.01 | 1.01 | 1.01 | 1.02 | 1.01 | 1.03 |
| 29 | 1.02 | 1.01 | 1.02 | 1.01 | 1.00 | 1.01 | 1.01 | 1.00 | 1.01 | 1.02 | 1.01 | 1.03 |
| 30 | 1.01 | 1.00 | 1.02 | 1.00 | 1.00 | 1.01 | 1.01 | 1.00 | 1.01 | 1.01 | 1.00 | 1.02 |
| 31 | 1.01 | 1.00 | 1.01 | 1.00 | 1.00 | 1.01 | 1.00 | 1.00 | 1.01 | 1.00 | 0.99 | 1.01 |
| 32 | 1.00 | 0.99 | 1.01 | 1.00 | 1.00 | 1.00 | 1.00 | 1.00 | 1.01 | 1.00 | 0.99 | 1.01 |
| 33 | 1.00 | 0.99 | 1.00 | 1.00 | 0.99 | 1.00 | 1.00 | 1.00 | 1.00 | 0.99 | 0.98 | 1.00 |
| 34 | 0.99 | 0.98 | 1.00 | 1.00 | 0.99 | 1.00 | 1.00 | 0.99 | 1.00 | 0.98 | 0.97 | 1.00 |
| 35 | 0.99 | 0.98 | 0.99 | 0.99 | 0.99 | 1.00 | 1.00 | 0.99 | 1.00 | 0.98 | 0.97 | 0.99 |
| 36 | 0.98 | 0.97 | 1.00 | 0.99 | 0.99 | 0.99 | 0.99 | 0.99 | 0.99 | 0.97 | 0.96 | 0.98 |
| 37 | 0.98 | 0.97 | 1.00 | 0.99 | 0.98 | 0.99 | 0.99 | 0.99 | 0.98 | 0.96 | 0.95 | 0.98 |
| 38 | 0.97 | 0.96 | 0.99 | 0.99 | 0.98 | 0.99 | 0.99 | 0.98 | 0.98 | 0.96 | 0.94 | 0.97 |
| 39 | 0.97 | 0.96 | 0.98 | 0.98 | 0.98 | 0.99 | 0.99 | 0.98 | 0.99 | 0.95 | 0.93 | 0.96 |
| 40 | 0.96 | 0.95 | 0.97 | 0.98 | 0.98 | 0.98 | 0.98 | 0.98 | 0.99 | 0.94 | 0.93 | 0.96 |
| 41 | 0.96 | 0.95 | 0.97 | 0.98 | 0.97 | 0.98 | 0.98 | 0.98 | 0.99 | 0.94 | 0.92 | 0.95 |
| 42 | 0.95 | 0.94 | 0.96 | 0.98 | 0.97 | 0.98 | 0.98 | 0.97 | 0.99 | 0.93 | 0.91 | 0.94 |

Note: Distributed lag non-linear logistic regression model adjusted for infant sex, maternal age, race, marital status, parity, maternal smoking, remoteness, areal level socioeconomic status, year, and calendar month of conception. P1-P99, first to 99^th^ centile of UTCI, Universal Thermal Climate Index in degree Celsius; OR, odds ratio; LCI and UCI, 95% lower and upper confidence intervals.

Table S2. The associations between maternal mean weekly UTCI exposures over twelve weeks preconception through to pregnancy and the odds of preeclampsia at various exposure thresholds with reference to median 14.2 ˚C in Western Australia, 2000–2015.

| Week | P1 (10.2 ˚C) | | | P5 (11.9 ˚C) | | | P95 (17.5 ˚C) | | | P99 (26.1 ˚C) | | |
| --- | --- | --- | --- | --- | --- | --- | --- | --- | --- | --- | --- | --- |
|  | OR | LCI | UCI | OR | LCI | UCI | OR | LCI | UCI | OR | LCI | UCI |
| -11 | 0.95 | 0.94 | 0.97 | 0.98 | 0.97 | 0.99 | 0.96 | 0.95 | 0.96 | 0.89 | 0.87 | 0.91 |
| -10 | 0.96 | 0.95 | 0.98 | 0.98 | 0.98 | 0.99 | 0.96 | 0.95 | 0.97 | 0.90 | 0.88 | 0.92 |
| -9 | 0.97 | 0.96 | 0.99 | 0.99 | 0.98 | 1.00 | 0.96 | 0.96 | 0.97 | 0.91 | 0.89 | 0.93 |
| -8 | 0.98 | 0.97 | 1.00 | 0.99 | 0.99 | 1.00 | 0.97 | 0.96 | 0.98 | 0.92 | 0.91 | 0.94 |
| -7 | 0.99 | 0.98 | 1.00 | 1.00 | 0.99 | 1.00 | 0.97 | 0.97 | 0.98 | 0.94 | 0.92 | 0.95 |
| -6 | 1.00 | 0.99 | 1.01 | 1.00 | 1.00 | 1.01 | 0.98 | 0.97 | 0.98 | 0.95 | 0.94 | 0.97 |
| -5 | 1.01 | 1.00 | 1.02 | 1.01 | 1.00 | 1.01 | 0.98 | 0.98 | 0.99 | 0.96 | 0.95 | 0.98 |
| -4 | 1.02 | 1.01 | 1.03 | 1.01 | 1.01 | 1.02 | 0.99 | 0.98 | 0.99 | 0.98 | 0.96 | 0.99 |
| -3 | 1.03 | 1.02 | 1.04 | 1.02 | 1.01 | 1.02 | 0.99 | 0.99 | 1.00 | 0.99 | 0.98 | 1.00 |
| -2 | 1.04 | 1.03 | 1.05 | 1.02 | 1.02 | 1.03 | 0.99 | 0.99 | 1.00 | 1.00 | 0.99 | 1.01 |
| -1 | 1.05 | 1.04 | 1.06 | 1.03 | 1.02 | 1.03 | 1.00 | 0.99 | 1.00 | 1.01 | 1.00 | 1.02 |
| 0 | 1.06 | 1.05 | 1.07 | 1.03 | 1.02 | 1.03 | 1.00 | 1.00 | 1.01 | 1.02 | 1.01 | 1.03 |
| 1 | 1.06 | 1.05 | 1.07 | 1.03 | 1.03 | 1.04 | 1.01 | 1.00 | 1.01 | 1.03 | 1.02 | 1.04 |
| 2 | 1.07 | 1.06 | 1.08 | 1.04 | 1.03 | 1.04 | 1.01 | 1.00 | 1.01 | 1.04 | 1.03 | 1.05 |
| 3 | 1.08 | 1.07 | 1.09 | 1.04 | 1.03 | 1.04 | 1.01 | 1.01 | 1.02 | 1.05 | 1.04 | 1.06 |
| 4 | 1.08 | 1.07 | 1.09 | 1.04 | 1.04 | 1.05 | 1.01 | 1.01 | 1.02 | 1.06 | 1.05 | 1.07 |
| 5 | 1.09 | 1.08 | 1.10 | 1.04 | 1.04 | 1.05 | 1.02 | 1.01 | 1.02 | 1.07 | 1.06 | 1.08 |
| 6 | 1.09 | 1.08 | 1.10 | 1.05 | 1.04 | 1.05 | 1.02 | 1.01 | 1.02 | 1.08 | 1.06 | 1.09 |
| 7 | 1.09 | 1.08 | 1.10 | 1.05 | 1.04 | 1.05 | 1.02 | 1.02 | 1.03 | 1.08 | 1.07 | 1.09 |
| 8 | 1.10 | 1.09 | 1.11 | 1.05 | 1.04 | 1.05 | 1.02 | 1.02 | 1.03 | 1.09 | 1.07 | 1.10 |
| 9 | 1.10 | 1.09 | 1.11 | 1.05 | 1.05 | 1.05 | 1.02 | 1.02 | 1.03 | 1.09 | 1.08 | 1.10 |
| 10 | 1.10 | 1.09 | 1.11 | 1.05 | 1.05 | 1.05 | 1.02 | 1.02 | 1.03 | 1.09 | 1.08 | 1.11 |
| 11 | 1.10 | 1.09 | 1.11 | 1.05 | 1.05 | 1.05 | 1.03 | 1.02 | 1.03 | 1.10 | 1.08 | 1.11 |
| 12 | 1.10 | 1.09 | 1.11 | 1.05 | 1.05 | 1.05 | 1.03 | 1.02 | 1.03 | 1.10 | 1.08 | 1.11 |
| 13 | 1.10 | 1.09 | 1.11 | 1.05 | 1.05 | 1.05 | 1.03 | 1.02 | 1.03 | 1.10 | 1.09 | 1.11 |
| 14 | 1.10 | 1.09 | 1.11 | 1.05 | 1.04 | 1.05 | 1.03 | 1.02 | 1.03 | 1.10 | 1.09 | 1.11 |
| 15 | 1.10 | 1.09 | 1.11 | 1.05 | 1.04 | 1.05 | 1.03 | 1.02 | 1.03 | 1.10 | 1.08 | 1.11 |
| 16 | 1.09 | 1.08 | 1.10 | 1.05 | 1.04 | 1.05 | 1.03 | 1.02 | 1.03 | 1.10 | 1.08 | 1.11 |
| 17 | 1.09 | 1.08 | 1.10 | 1.05 | 1.04 | 1.05 | 1.03 | 1.02 | 1.03 | 1.09 | 1.08 | 1.11 |
| 18 | 1.09 | 1.08 | 1.10 | 1.04 | 1.04 | 1.05 | 1.02 | 1.02 | 1.03 | 1.09 | 1.08 | 1.10 |
| 19 | 1.08 | 1.07 | 1.09 | 1.04 | 1.04 | 1.05 | 1.02 | 1.02 | 1.03 | 1.09 | 1.08 | 1.10 |
| 20 | 1.08 | 1.07 | 1.09 | 1.04 | 1.03 | 1.04 | 1.02 | 1.02 | 1.03 | 1.08 | 1.07 | 1.09 |
| 21 | 1.07 | 1.06 | 1.08 | 1.04 | 1.03 | 1.04 | 1.02 | 1.02 | 1.02 | 1.08 | 1.07 | 1.09 |
| 22 | 1.07 | 1.06 | 1.08 | 1.03 | 1.03 | 1.04 | 1.02 | 1.01 | 1.02 | 1.07 | 1.06 | 1.08 |
| 23 | 1.06 | 1.05 | 1.07 | 1.03 | 1.03 | 1.03 | 1.02 | 1.01 | 1.02 | 1.07 | 1.06 | 1.08 |
| 24 | 1.06 | 1.05 | 1.06 | 1.03 | 1.02 | 1.03 | 1.02 | 1.01 | 1.02 | 1.06 | 1.05 | 1.07 |
| 25 | 1.05 | 1.04 | 1.06 | 1.02 | 1.02 | 1.03 | 1.01 | 1.01 | 1.02 | 1.06 | 1.04 | 1.07 |
| 26 | 1.04 | 1.03 | 1.05 | 1.02 | 1.02 | 1.02 | 1.01 | 1.01 | 1.02 | 1.05 | 1.04 | 1.06 |
| 27 | 1.04 | 1.03 | 1.04 | 1.02 | 1.01 | 1.02 | 1.01 | 1.00 | 1.01 | 1.04 | 1.03 | 1.05 |
| 28 | 1.03 | 1.02 | 1.03 | 1.01 | 1.01 | 1.02 | 1.01 | 1.00 | 1.01 | 1.03 | 1.02 | 1.04 |
| 29 | 1.02 | 1.01 | 1.03 | 1.01 | 1.01 | 1.01 | 1.00 | 1.00 | 1.01 | 1.03 | 1.01 | 1.04 |
| 30 | 1.01 | 1.01 | 1.02 | 1.01 | 1.00 | 1.01 | 1.00 | 1.00 | 1.01 | 1.02 | 1.01 | 1.03 |
| 31 | 1.00 | 1.00 | 1.01 | 1.00 | 1.00 | 1.01 | 1.00 | 0.99 | 1.00 | 1.01 | 1.00 | 1.02 |
| 32 | 1.00 | 0.99 | 1.00 | 1.00 | 0.99 | 1.00 | 1.00 | 0.99 | 1.00 | 1.00 | 0.99 | 1.01 |
| 33 | 0.99 | 0.98 | 1.00 | 0.99 | 0.99 | 1.00 | 0.99 | 0.99 | 1.00 | 0.99 | 0.98 | 1.00 |
| 34 | 0.98 | 0.97 | 0.99 | 0.99 | 0.99 | 0.99 | 0.99 | 0.98 | 1.00 | 0.98 | 0.97 | 1.00 |
| 35 | 0.97 | 0.96 | 0.98 | 0.98 | 0.98 | 0.99 | 0.99 | 0.98 | 0.99 | 0.97 | 0.96 | 0.99 |
| 36 | 0.96 | 0.95 | 0.97 | 0.99 | 0.98 | 0.98 | 0.98 | 0.98 | 0.99 | 0.96 | 0.95 | 0.98 |
| 37 | 0.95 | 0.95 | 0.96 | 0.98 | 0.97 | 0.98 | 0.98 | 0.98 | 0.99 | 0.96 | 0.94 | 0.97 |
| 38 | 0.95 | 0.94 | 0.96 | 0.97 | 0.97 | 0.98 | 0.98 | 0.97 | 0.99 | 0.95 | 0.93 | 0.96 |
| 39 | 0.94 | 0.93 | 0.95 | 0.97 | 0.96 | 0.97 | 0.98 | 0.97 | 0.98 | 0.94 | 0.92 | 0.95 |
| 40 | 0.93 | 0.92 | 0.94 | 0.96 | 0.96 | 0.97 | 0.97 | 0.96 | 0.98 | 0.93 | 0.91 | 0.95 |
| 41 | 0.92 | 0.91 | 0.93 | 0.96 | 0.95 | 0.96 | 0.97 | 0.96 | 0.98 | 0.92 | 0.90 | 0.94 |
| 42 | 0.91 | 0.90 | 0.92 | 0.95 | 0.95 | 0.96 | 0.97 | 0.96 | 0.97 | 0.91 | 0.89 | 0.93 |

Note: Distributed lag non-linear logistic regression model adjusted for infant sex, maternal age, race, marital status, parity, maternal smoking, remoteness, areal level socioeconomic status, year, and calendar month of conception. P1-P99, first to 99^th^ centile of UTCI, Universal Thermal Climate Index in degree Celsius; OR, odds ratio; LCI and UCI, 95% lower and upper confidence intervals.

Table S3. Simultaneous and separate models for trimester-specific associations between maternal UTCI exposures and the odds of gestational hypertension and preeclampsia at various exposure thresholds with reference to median 14.2 ˚C in Western Australia, 2000–2015.

| Model | Exposure periods |  | Gestational hypertension | | | Preeclampsia | | |
| --- | --- | --- | --- | --- | --- | --- | --- | --- |
|  |  | UTCI centile | OR | LCI | UCI | OR | LCI | UCI |
| Simultaneous* | First trimester | P1 | 0.84 | 0.72 | 0.99 | 1.51 | 1.27 | 1.80 |
|  |  | P5 | 0.92 | 0.80 | 1.04 | 1.34 | 1.16 | 1.55 |
|  |  | P95 | 1.35 | 1.22 | 1.50 | 0.98 | 0.87 | 1.10 |
|  |  | P99 | 1.15 | 0.95 | 1.39 | 0.94 | 0.76 | 1.16 |
|  | Second trimester | P1 | 1.15 | 0.99 | 1.34 | 1.17 | 0.99 | 1.39 |
|  |  | P5 | 1.11 | 0.98 | 1.26 | 1.14 | 0.99 | 1.31 |
|  |  | P95 | 1.21 | 1.07 | 1.36 | 1.16 | 1.01 | 1.32 |
|  |  | P99 | 0.97 | 0.77 | 1.22 | 1.04 | 0.81 | 1.35 |
|  | Third trimester | P1 | 1.25 | 1.11 | 1.41 | 2.07 | 1.83 | 2.34 |
|  |  | P5 | 1.19 | 1.06 | 1.32 | 1.60 | 1.42 | 1.81 |
|  |  | P95 | 1.25 | 1.13 | 1.38 | 1.33 | 1.19 | 1.49 |
|  |  | P99 | 1.02 | 0.84 | 1.24 | 1.71 | 1.40 | 2.09 |
| Separate* | First trimester | P1 | 0.76 | 0.66 | 0.89 | 1.14 | 0.99 | 1.31 |
|  |  | P5 | 0.90 | 0.82 | 1.00 | 1.07 | 0.96 | 1.20 |
|  |  | P95 | 1.27 | 1.17 | 1.39 | 1.01 | 0.92 | 1.12 |
|  |  | P99 | 1.11 | 0.98 | 1.25 | 0.91 | 0.80 | 1.04 |
|  | Second trimester | P1 | 0.93 | 0.82 | 1.05 | 1.09 | 0.95 | 1.25 |
|  |  | P5 | 0.93 | 0.84 | 1.02 | 1.02 | 0.91 | 1.13 |
|  |  | P95 | 1.20 | 1.10 | 1.31 | 1.00 | 0.90 | 1.10 |
|  |  | P99 | 1.11 | 0.99 | 1.26 | 0.99 | 0.87 | 1.12 |
|  | Third trimester | P1 | 1.24 | 1.11 | 1.37 | 2.00 | 1.79 | 2.23 |
|  |  | P5 | 1.19 | 1.08 | 1.31 | 1.56 | 1.40 | 1.73 |
|  |  | P95 | 1.25 | 1.15 | 1.35 | 1.22 | 1.12 | 1.34 |
|  |  | P99 | 1.28 | 1.13 | 1.44 | 1.37 | 1.21 | 1.56 |

Note: *Simultaneous= three trimester-specific average exposures were included simultaneously in one model; Separate= three separate trimester-specific average exposures were analysed in individual models. Standard non-linear logistic regression model adjusted for infant sex, maternal age, race, marital status, parity, maternal smoking, remoteness, areal level socioeconomic status, year, and calendar month of conception. P1-P99, first to 99^th^ centile of UTCI; UTCI, Universal Thermal Climate Index in degree Celsius; OR, odds ratio; LCI and UCI, 95% lower and upper confidence intervals.

**Figures**

Total number of pregnancies in Midwives

Notifications System between 1^st^ January 2000 and 31^st^ December 2015 (n = 467,760)

Excluded missing SA1 (n 3,4841=)

Pregnancies with SA1 address

(n = 432,919)

Excluded missing conception date (n= 1017)

Pregnancies with date of conception

(n = 431,902)

Excluded multiple births

(n= 7,475)

Singleton pregnancies (n= 425,444)

Excluded gestational age outside 20-42 (n=530)

Gestational age 20-42 weeks

(n= 424914)

Excluded Mothers aged > 45 years (n= 269)

Maternal age ≤ 45 years (n= 424645)

Excluded births conceived before 14^th^ August 1999 (n= 8,099)

Conception date 20 weeks before cohort started (n = 416546)

Excluded births conceived after 12^th^ March 2015 (n = 1,450)

Conception date 42 weeks before cohort ended across (n = 415,096)

Excluded incorrect SA1 to assign exposure (n=5)

Included sample cohort (n = 415,091)

Figure S1. Flow chart for selecting the eligible pregnancies or births included in this study, Western Australia, 2000-2015. Note: SA1, statistical area level 1.

| Gestational hypertension  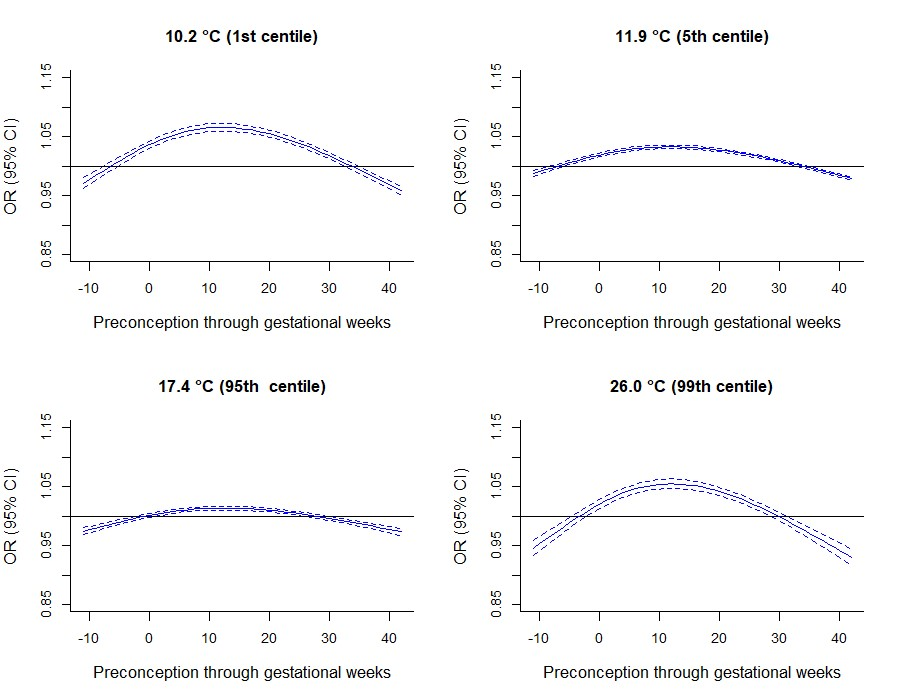 |
| --- |
| Preeclampsia  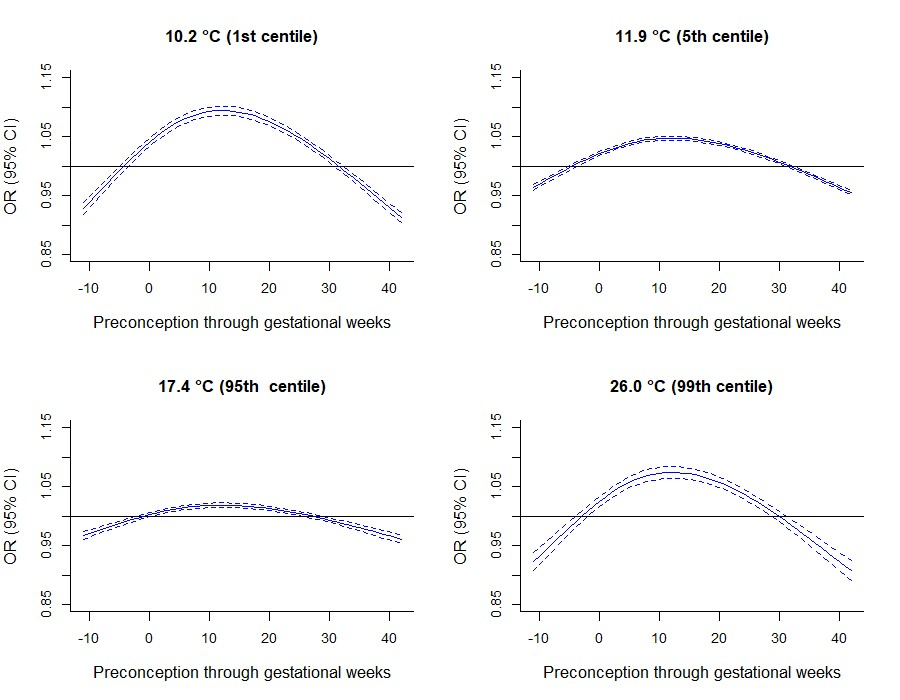 |

Figure S2. Weekly-specific UTCI for 12 weeks preconception (-11 to 0) through to gestational week (1 to 42) and the unadjusted odds of gestational hypertension at different thresholds of UTCI with reference to the mean of 14.5 ◦C instead of the median of 14.2 ◦C. The blue solid lines represent point estimates, and the broken lines represent 95% confidence intervals. All models included only *crossbasis* matrix of UTCI without adjusting for any covariates. Note: OR, odds ratio; CI, confidence interval; UTCI, Universal Thermal Climate Index.

| Early-onset gestational hypertension  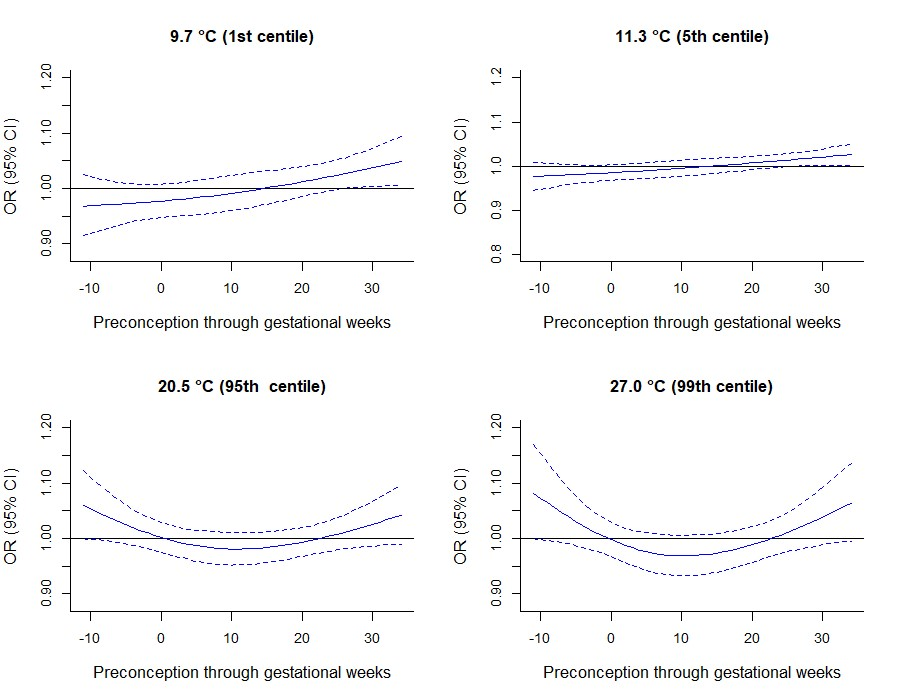 |
| --- |
| Late-onset gestational hypertension  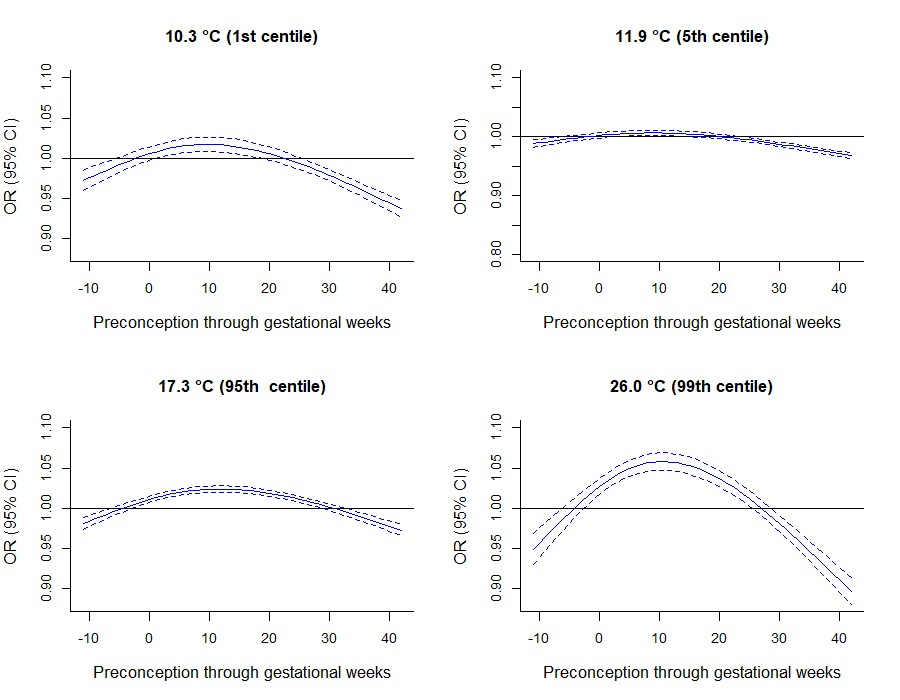 |

Figure S3. Weekly-specific UTCI for 12 weeks preconception (-11 to 0) through to gestational week (1 to 34 and 1 to 42) and the odds of early and late onsets of gestational hypertension at different thresholds of UTCI with reference to the median of 14.2 ◦C. The blue solid lines represent point estimates, and the broken lines represent 95% confidence intervals. All models were adjusted for infant sex, maternal age, race or ethnicity, marital status, smoking status, parity, remoteness, socioeconomic status, and year and month of conception. Note: OR, odds ratio; CI, confidence interval; UTCI, Universal Thermal Climate Index.

| Early-onset preeclampsia  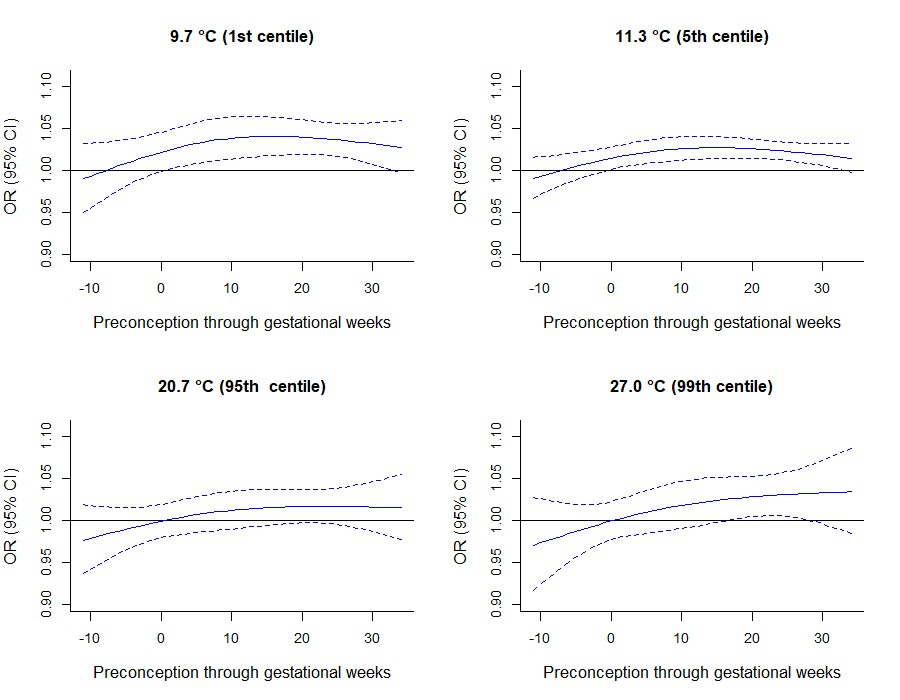 |
| --- |
| Late-onset preeclampsia  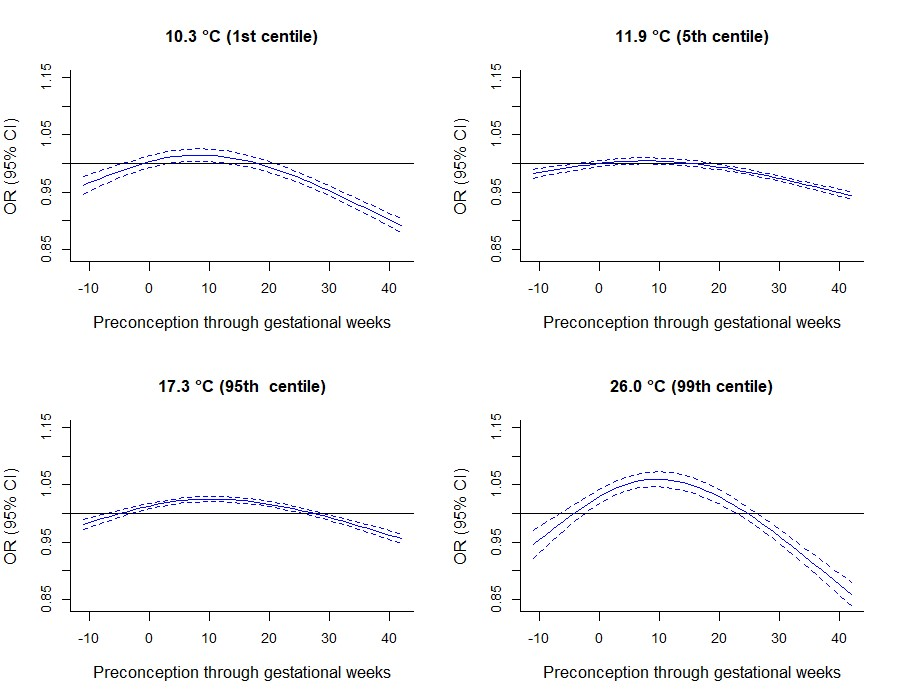 |

Figure S4. Weekly-specific UTCI for 12 weeks preconception (-11 to 0) through to gestational week (1 to 34 and 1 to 42) and the odds of early and late onsets of preeclampsia at different thresholds of UTCI with reference to the median of 14.2 ◦C. The blue solid lines represent point estimates, and the broken lines represent 95% confidence intervals. All models were adjusted for infant sex, maternal age, race or ethnicity, marital status, smoking status, parity, remoteness, socioeconomic status, and year and month of conception. Note: OR, odds ratio; CI, confidence interval; UTCI, Universal Thermal Climate Index.

| Gestational hypertension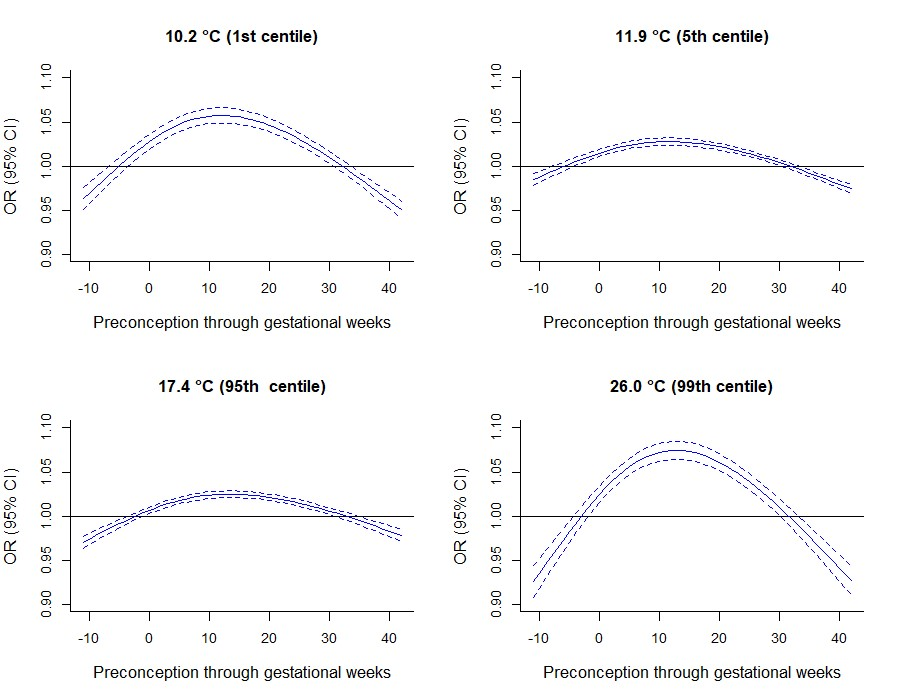 |
| --- |
| Preeclampsia  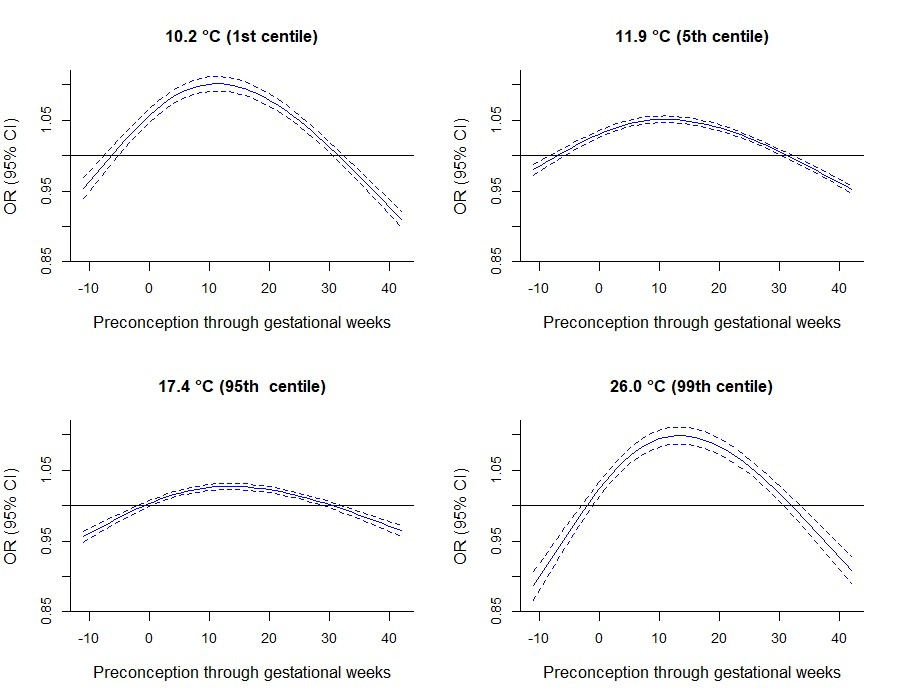 |

Figure S5. Weekly-specific UTCI for 12 weeks preconception (-11 to 0) through to gestational week (1 to 42) and the odds of gestational hypertension at different thresholds of UTCI with reference to the mean of 14.5 ◦C instead of the median of 14.2 ◦C. The blue solid lines represent point estimates, and the broken lines represent 95% confidence intervals. All models were adjusted for infant sex, maternal age, race or ethnicity, marital status, smoking status, parity, remoteness, socioeconomic status, and year and month of conception. Note: OR, odds ratio; CI, confidence interval; UTCI, Universal Thermal Climate Index.

| Gestational hypertension  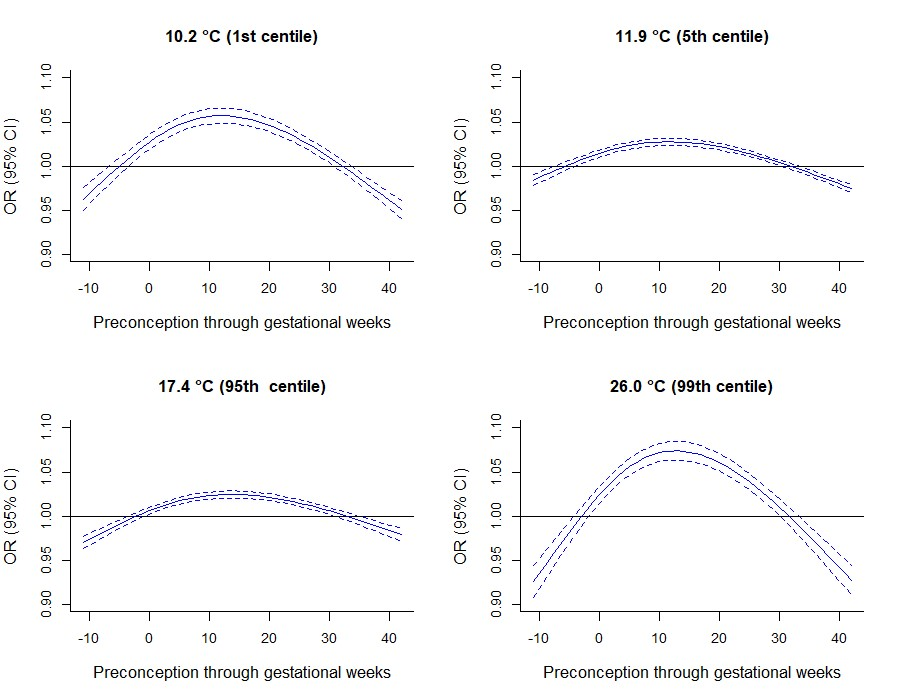 |
| --- |
| Preeclampsia  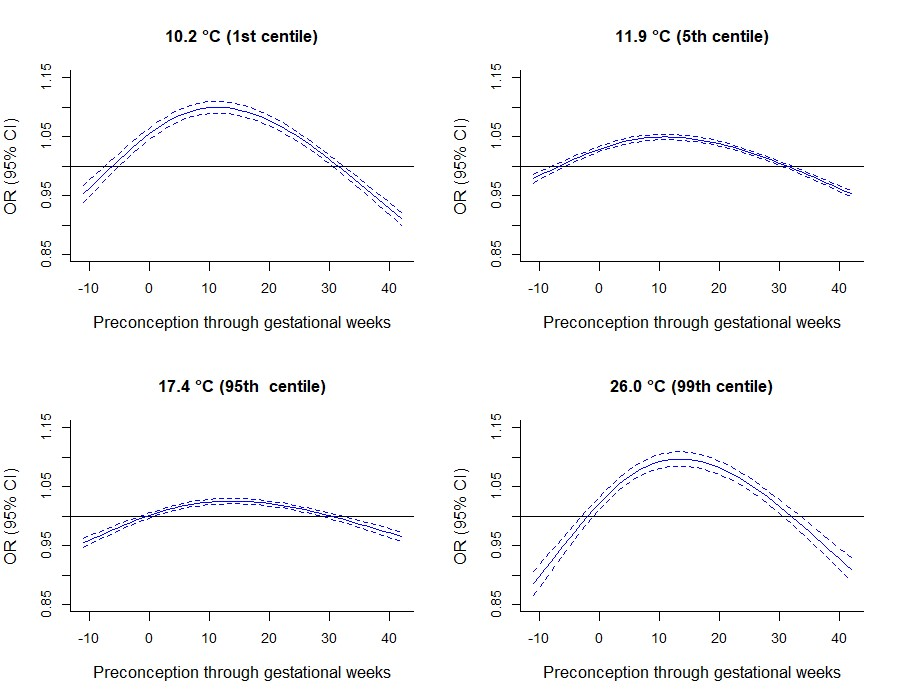 |

Figure S6. Weekly-specific UTCI for 12 weeks preconception (-11 to 0) through to gestational week (1 to 42) and the odds of gestational hypertension at different thresholds of UTCI with reference to the median of 14.2 ◦C. The blue solid lines represent point estimates, and the broken lines represent 95% confidence intervals. Maternal age was adjusted as a categorical variable (≤19, 20-34, ≥35) instead of natural splines of a continuous variable. All models were adjusted for infant sex, maternal age, race or ethnicity, marital status, smoking status, parity, remoteness, socioeconomic status, and year and month of conception. Note: OR, odds ratio; CI, confidence interval; UTCI, Universal Thermal Climate Index.

| Gestational hypertension  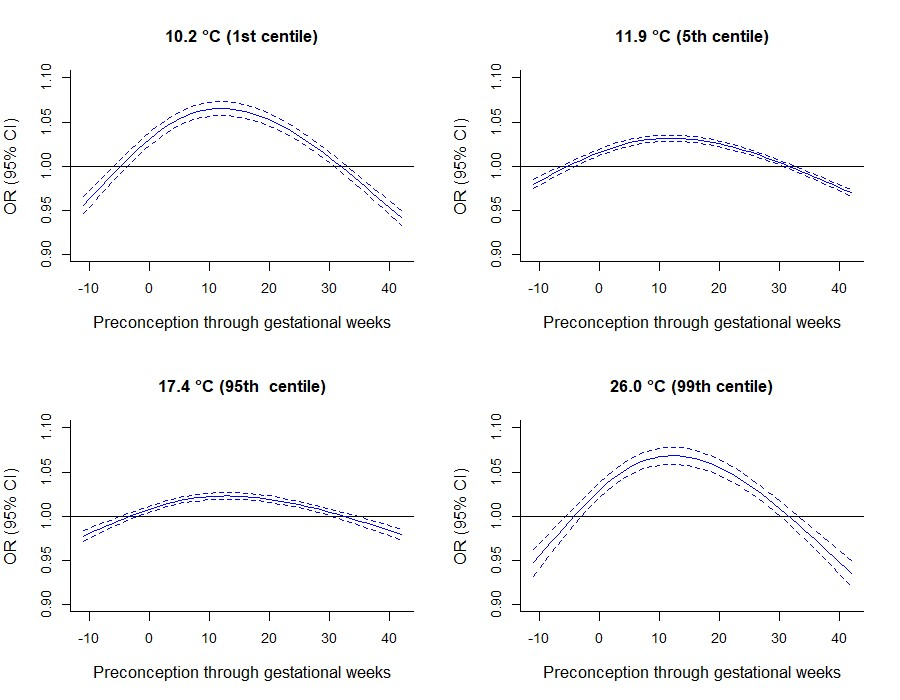 |
| --- |
| Preeclampsia  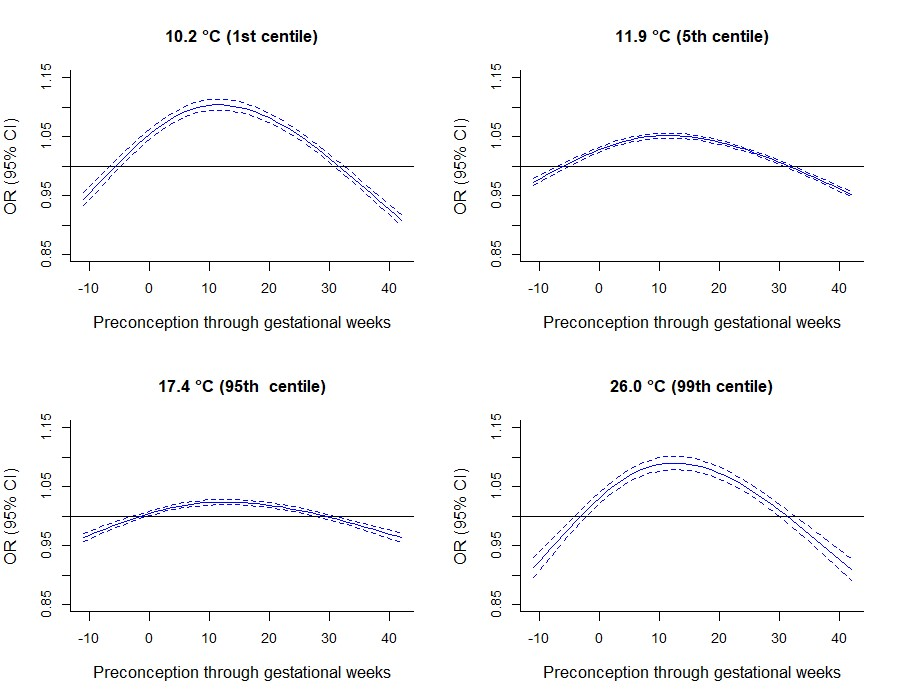 |

Figure S7. Weekly-specific UTCI for 12 weeks preconception (-11 to 0) through to gestational week (1 to 42) and the odds of gestational hypertension at different thresholds of UTCI with reference to the median of 14.2 ◦C. The blue solid lines represent point estimates, and the broken lines represent 95% confidence intervals. The season of conception was adjusted as four-season categories (autumn, winter, spring, summer) instead of calendar months (1 to 12). All models were adjusted for infant sex, maternal age, race or ethnicity, marital status, smoking status, parity, remoteness, socioeconomic status, and year and month of conception. Note: OR, odds ratio; CI, confidence interval; UTCI, Universal Thermal Climate Index.

| Gestational hypertension  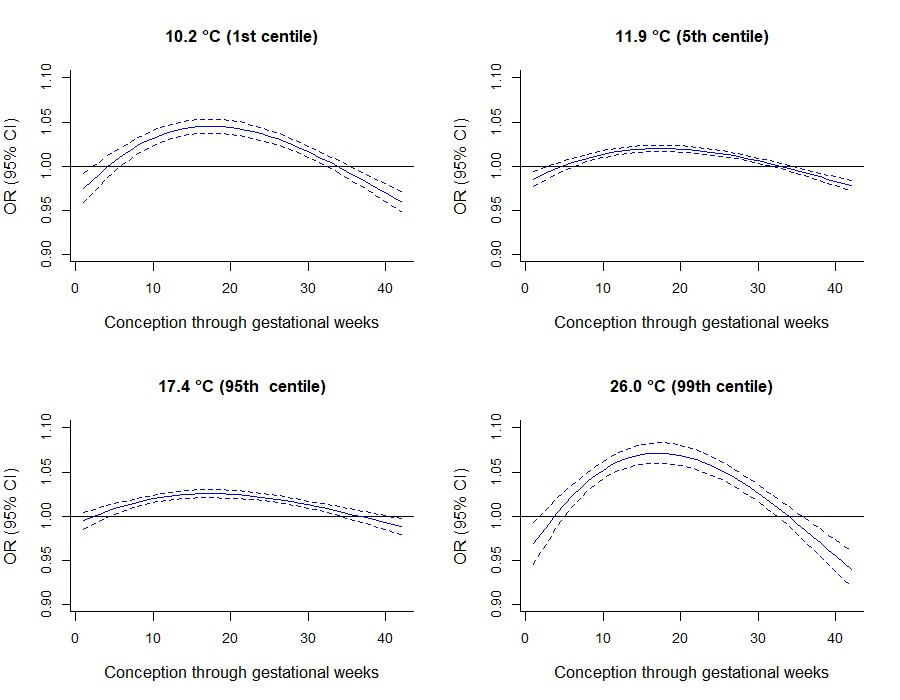 |
| --- |
| Preeclampsia  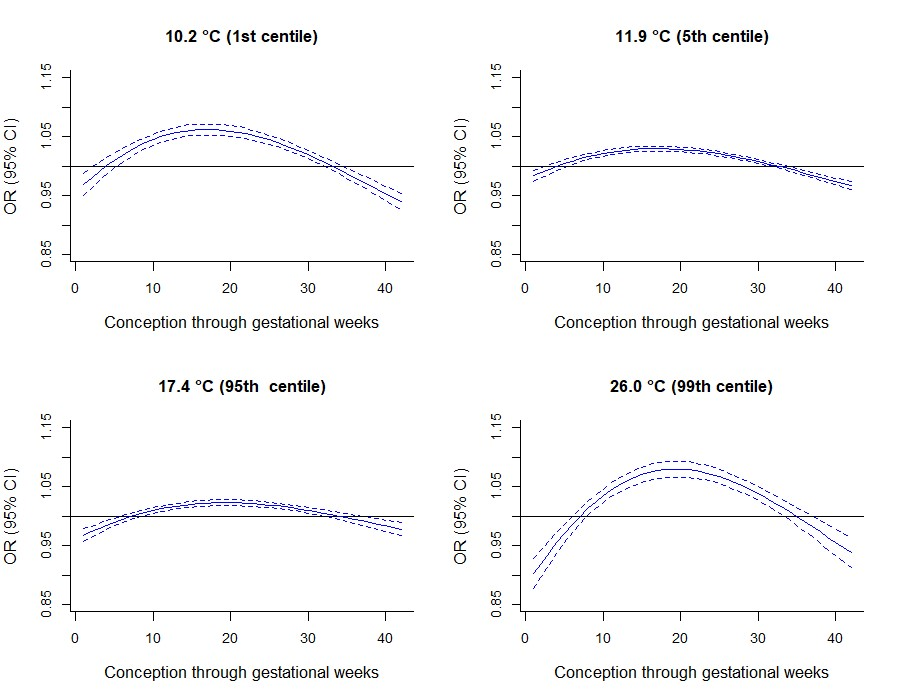 |

Figure S8. Weekly-specific UTCI from conception through gestational week and the odds of gestational hypertension at different thresholds of UTCI with reference to the median of 14.2 ◦C. The blue solid lines represent point estimates, and the broken lines represent 95% confidence intervals. All models were adjusted for infant sex, maternal age, race or ethnicity, marital status, smoking status, parity, remoteness, socioeconomic status, and year and month of conception. Note: OR, odds ratio; CI, confidence interval; UTCI, Universal Thermal Climate Index.

| Gestational hypertension  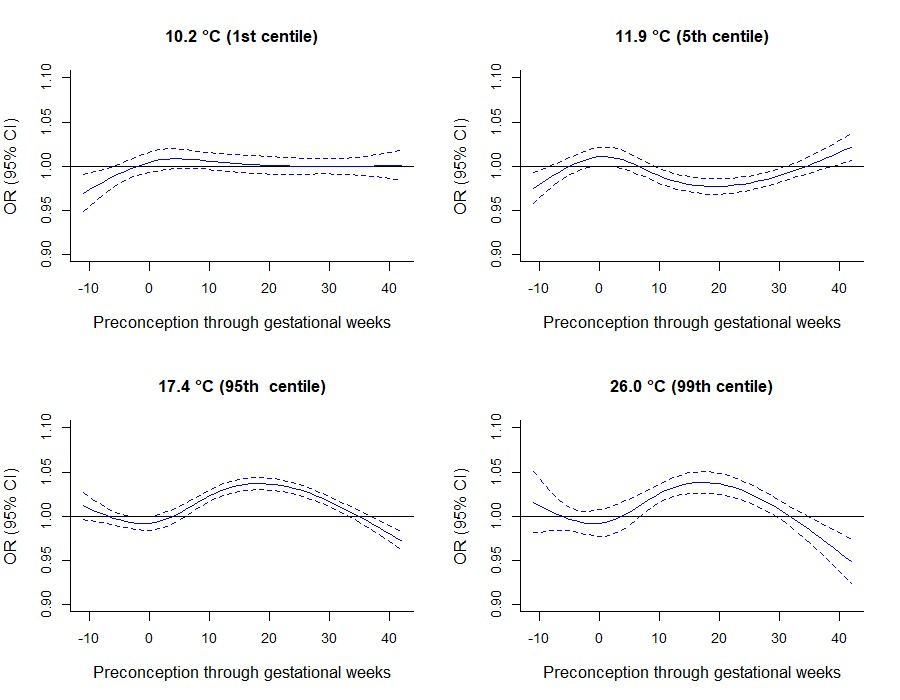 |
| --- |
| Preeclampsia  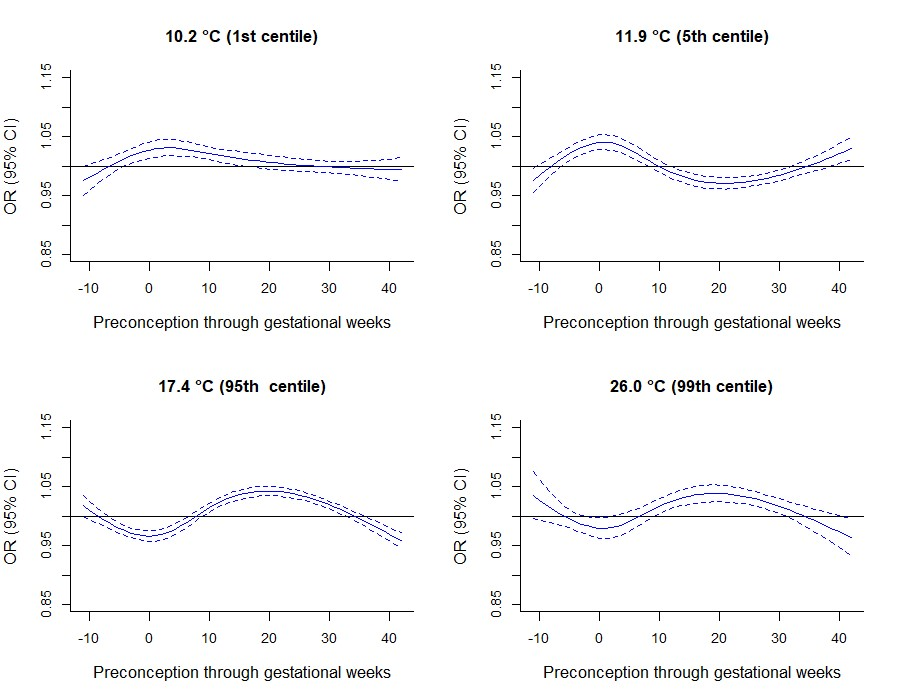 |

Figure S9. Weekly-specific UTCI for 12 weeks preconception (-11 to 0) through to gestational week (1 to 42) and the odds of gestational hypertension at different thresholds of UTCI with reference to the median of 14.2 ◦C. The blue solid lines represent point estimates, and the broken lines represent 95% confidence intervals. The *dfs* in the natural cubic spline in the cross-basis function were changed to 5 and 4 *dfs* for exposure-response and exposure period-response associations, respectively. All models were adjusted for infant sex, maternal age, race or ethnicity, marital status, smoking status, parity, remoteness, socioeconomic status, and year and month of conception. Note: OR, odds ratio; CI, confidence interval; UTCI, Universal Thermal Climate Index.
